# Facile Synthesis of Sulfur-Containing Functionalized Disiloxanes with Nonconventional Fluorescence by Thiol–Epoxy Click Reaction

**DOI:** 10.3390/ijms24097785

**Published:** 2023-04-24

**Authors:** Jing Tang, Shengyu Feng, Dengxu Wang

**Affiliations:** 1 Institute of Novel Semiconductors, State Key Laboratory of Crystal Materials, Shandong University, Jinan 250100, China; 2 National Engineering Research Center for Colloidal Materials & Key Laboratory of Special Functional Aggregated Materials, Ministry of Education, Shandong Key Laboratory of Advanced Organosilicon Materials and Technologies, School of Chemistry and Chemical Engineering, Shandong University, Jinan 250100, China

**Keywords:** thiol–epoxy reaction, functionalized disiloxanes, organosilicon synthetic methodology, nonconventional fluorescence

## Abstract

Herein, a series of novel sulfur-containing functionalized disiloxanes based on a low-cost and commercially available material, i.e., 1,3-bis(3-glycidoxypropyl)-1,1,3,3-tetramethyldisiloxane, and various thiol compounds were prepared by thiol–epoxy click reaction. It was found that both lithium hydroxide (LiOH) and tetrabutylammonium fluoride (TBAF) have high catalytic activity after optimizing the reaction condition, and the reaction can be carried out with high yields, excellent regioselectivity, mild reaction condition, and good tolerance of functional groups. These compounds exhibit excellent nonconventional fluorescence due to the formation of coordination bonds between Si atoms and heteroatoms (e.g., S or N) and can emit blue fluorescence upon ultraviolet (UV) irradiation. These results demonstrate that the thiol–epoxy click reaction could promisingly act as an efficient organosilicon synthetic methodology to construct various organosilicon materials with novel structures and functionality, and thus their application scope will be significantly expanded.

## 1. Introduction

In recent years, intense efforts have been devoted to developing new organosilicon materials because of their unique properties and indispensable roles in emerging areas, such as aerospace, new energy, electronics, and biomedicine [1,2,3,4,5]. However, the development lags far behind the increasing requirements. One crucial reason is that organosilicon synthetic methodologies are relatively few compared to numerous synthetic strategies to build carbon-based materials. Up to now, three traditional methodologies, including direct synthesis, hydrolysis and polycondensation, and hydrosilylation reactions, are still the most widely used techniques in the laboratory and in industry [6,7,8,9], although these traditional methodologies were found more than 80 years ago. Thus, developing new efficient synthetic methodologies to design and construct organosilicon materials is still highly desirable.

Thanks to the well-developed and emerging organic synthetic methodologies, diversified organosilicon materials with novel structures and functionality have been designed, prepared, and new applications found, and thus organosilicon synthetic methodologies are increasingly growing [10,11,12,13,14,15,16]. Click reactions have attracted specific attention because of their intriguing features, such as mild reaction conditions, fast speed, high yield, and brilliant tolerance of functional groups [17,18,19]. The azide–alkyne reaction [20], thiol–ene reaction [21,22], and aza-Michael addition reaction [23,24] with the “click” feature have all been widely used to prepare organosilicon compounds [21], functionalized polysiloxanes [20,25,26,27], and silicone elastomers [28] by us and other groups, and thus have become mature or semi-mature organosilicon synthetic methodologies. From the standpoint of existing raw materials, these strategies require azide- (or alkyl), thiol-, vinyl-, and amine-containing silanes or (poly)siloxanes, most of which have relatively high costs except vinyl-containing materials. However, epoxy-based materials (e.g., epoxy-containing disiloxane and polysiloxanes), which have a much lower cost than thiol- or amine-based ones, have not been fully exploited. It is of great interest to develop an organosilicon synthetic methodology using epoxy groups.

Herein, we introduce the thiol–epoxy reaction as the new organosilicon synthetic methodology and choose the preparation of functionalized disiloxanes as the representative examples. The selection of the thiol–epoxy reaction is motivated by its click feature and wide usage in the construction of functional molecules and polymers [29,30]. Although this reaction has been used to prepare some organosilicon materials [31], such as silsesquioxane-based materials [32,33,34,35,36], silicone/epoxy composites [37,38], and epoxy/silanes hybrid coating [39], exploration in this area is still limited and systematical investigation is rare. Moreover, the nonconventional properties of organosilicon materials constructed by this reaction have still not been studied despite the presence of unique S→Si coordination bonds within the structures. Thus, a systematical investigation on the synthesis of new sulfur-containing functionalized disiloxanes by the thiol–epoxy reaction is reported in the present study. The catalytic system and reaction condition for the synthesis of organosilicon compounds were optimized by studying the effects of catalysts, substituents, reaction temperature, and reaction times on the reaction efficiency. Using the optimal reaction, eleven new functionalized disiloxanes were prepared and their nonconventional fluorescent properties were explored.

## 2. Results and Discussion

### 2.1. Optimization of Reaction Condition

**Scheme 1:**
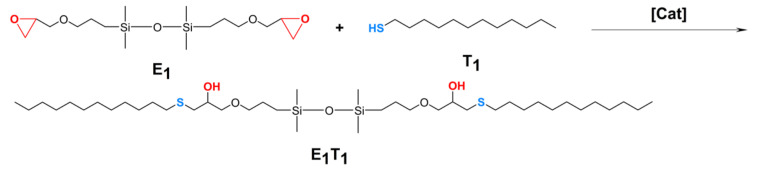
The model reaction between 1,3-bis(3-glycidoxypropyl)tetramethyldisiloxane (E_1_) and 1-dodecanethiol (T_1_).

The thiol–epoxy reaction is considered a click reaction [40] when using the base as the catalyst, and previous reports proved that tetrabutylammonium fluoride (TBAF) and lithium hydroxide (LiOH) were the most efficient catalysts [41]. When this reaction was used to prepare organosilicon materials, the present sulfur-containing functionalized disiloxanes, the base catalyst is required to evaluate the suitability because of the sensitivity of Si–O or Si–C bonds under basic conditions. Thus a model reaction between 1,3-bis(3-glycidoxypropyl)tetramethyldisiloxane (E_1_) and 1-dodecanethiol (T_1_) was conducted to find out the optimal condition using LiOH or TBAF as catalysts under solvent-free condition or in tetrahydrofuran (THF) (Scheme 1) [41]. Table 1 demonstrates the thiol–epoxy reaction data of E_1_ and T_1_.

LiOH was initially used as the catalyst (5 mol%) without solvent and with a reaction time of 6 h at room temperature. The conversion rate of the epoxy group is 63.1% (Table 1, entry 1). It is a good sign that prolonging the reaction time to 12 h led to the quantitative conversion of the epoxy group (Table 1, entry 2). However, when the amount of catalyst was reduced to 3 mol% and 1 mol%, the conversion rates were significantly decreased to 80.3% and 39.8%, respectively (Table 1, entries 3–4). Then TBAF was used as a catalyst in THF. It was found that the conversion rate of the epoxy group was 81.3% with 8 mol% of TBAF and a reaction time of 12 h at room temperature (Table 1, entry 5). When the temperature was increased to 50 °C, the epoxy groups could be quantitatively consumed (Table 1, entry 6). Then, the amount of catalyst was reduced to 5 mol%, 3 mol%, 1 mol%, and 0.8 mol% under the same condition (Table 1, entries 7–10) and it was found that 3 mol% of catalyst could still provide high catalytic activity with a high conversion rate of the epoxy group (98.6%) (Table 1, entry 8). Based on this finding, the reaction time was further optimized with 3 mol% of catalyst loading (Table 1, entries 11–12). The results demonstrated that 6 h was sufficient to achieve almost quantitative conversion (Table 1, entry 11).

In addition, no obvious by-products, which may be generated by the cleavage of Si–O or Si–C bonds, were found in the final products using TBAF and LiOH as the catalysts, as evidenced by the high yields (98% and 96% for entries 2 and 11) after purification and the ^29^Si NMR spectra of the reaction mixture, in which no peaks in the ranges of −30 ppm to −20 ppm and 20 ppm to 40 ppm, which are assigned to the silicon atoms from Si–OH or Si-F bonds, respectively, were observed during the reaction (Figure S1). These results indicate that both TBAF and LiOH can act as efficient catalysts for the synthesis of new functionalized disiloxanes. It is known that LiOH is soluble in water but insoluble in most of organic solvents; thus, it is considered a heterogeneous catalyst [30]. Although it can efficiently catalyze this reaction of E_1_T_1_ without the solvent, its catalytic activity is relatively low when thiol compounds (e.g., methimazole) appear as the solid, and thus the condition (e.g., additional solvents) needs to be further optimized [30]. In contrast, this possibility will not occur when selecting TBAF in THF because THF can dissolve most thiol compounds. In addition, the alkalinity of TBAF is weaker than LiOH, and the residual LiOH in the system may cause the instability of the products (e.g., crosslinking networks) [42]. Thus, considering the universality of TBAF in THF, herein we choose it for further synthesis of various sulfur-containing functionalized disiloxanes.

### 2.2. Synthesis and Characterization of Sulfur-Containing Functionalized Disiloxanes

**Scheme 2:**
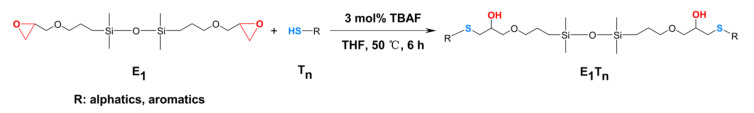
Synthetic routes of sulfur-containing functionalized disiloxanes from 1,3-bis(3-glycidoxypropyl)tetramethyldisiloxane (E_1_) and various thiol compounds (T_n_).

The capability of the optimized reaction condition, that is TBAF (3 mol%) in THF at 50 °C for 6 h, was tested by preparing sulfur-containing functionalized disiloxanes from E_1_ and various thiol compounds (Scheme 2). The results revealed that eleven new disiloxanes were successfully achieved with excellent conversion rates of epoxy groups (≥99%) and isolated yields (>89%) and high regioselectivity (Table 2, entries 1–11) regardless of aliphatic thiols (e.g., 1-butanethiol, a yield of 95.5%, Table 2, entry 2) or aromatic thiols (e.g., 3-methylbenzenethiol, a yield of 96.5%, Table 2, entry 9). As expected, in using aromatic thiols, the products can be generated with high yields, even shortening the reaction times due to their stronger nucleophile ability of the formed thiolate anion than aliphatic thiols [30,43]. For example, when 3-methylbenzenethiol (T_9_) reacted with E_1_, 3 h was sufficient to afford a nearly quantitative conversion of the epoxy group (Table 2, entry 16). In addition, the yield of E_1_T_5_ (Table 2, entry 5) was slightly lower than that of E_1_T_2_ (Table 2, entry 2), and this finding could be explained by higher steric hindrance of 3-methyl-2-butanethiol (T_5_) than 1-butanethiol (T_2_). This speculation can also explain the fact that E_1_T_2_ and E_1_T_4_ can also be afforded with high yields, even shortening the reaction time to 3 h or 5 h (Table 2, entries 12 and 14), in comparison to the requirement of 6 h for E_1_T_1_ (Table 2, entry 1), concerning the higher steric hindrance of T_1_ than T_2_ and T_4_. These results demonstrated that the electronic effect and steric hindrance of substituent groups influenced the activity of thiol groups, resulting in the different reactivity of thiol compounds and further different yields.

**Figure 1:**
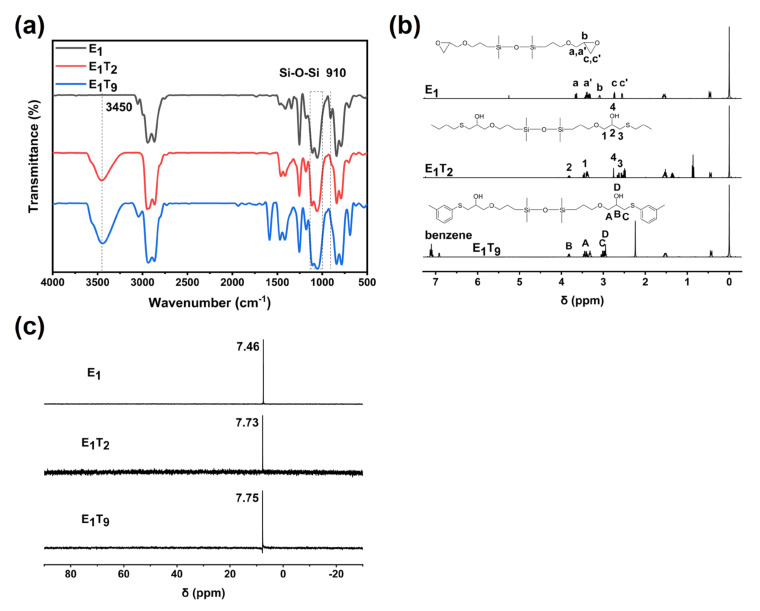
(**a**) FT-IR spectra of epoxy-containing groups disiloxane E_1_ and functionalized disiloxanes E_1_T_2_, E_1_T_9_. (**b**) ^1^H NMR spectrum of E_1_, E_1_T_2_, and E_1_T_9_ in CDCl_3_. (**c**) ^29^Si NMR spectrum of E_1_, E_1_T_2_, and E_1_T_9_ in CDCl_3_.

The successful achievement of these sulfur-containing functionalized disiloxanes can be determined by FT-IR, ^1^H/^13^C/^29^Si NMR, HR-MS, and elemental analysis. Considering their similar structures, E_1_T_2_ and E_1_T_9_ were selected as the representative examples (the original spectra of all the products can be found in the Figures S2−S19 of the Supporting Information). Figure 1a illustrates the IR spectrum of E_1_, E_1_T_2_, and E_1_T_9_. After the reaction, the peaks assigned to the epoxy group at 910 cm^−1^ disappeared in the products E_1_T_2_ and E_1_T_9_, while a new strong and broad peak attributed to the generated β-hydroxyl group at 3464 cm^−1^ was observed. Figure 1b illustrates the ^1^H NMR of E_1_, E_1_T_2_, and E_1_T_9_, and the assignments of the characteristic protons. The peaks assigned to epoxy protons at 2.55 ppm (H^c’^), 2.74 ppm (H^c^), and 3.09 ppm (H^b^) in E_1_ completely disappeared in E_1_T_2_ and E_1_T_9_, while two new signals at 2.76 ppm and 3.82 ppm, which are attributed to the protons from β-hydroxyl groups (H^4^ in E_1_T_2_ and H^D^ in E_1_T_9_) and −CH groups adjacent to it (H^2^ in E_1_T_2_ and H^B^ in E_1_T_9_), were observed (Figure 1b). In the ^29^Si NMR, a single peak was found at 7.73 ppm and 7.75 ppm for E_1_T_2_ and E_1_T_9_, in comparison to a single peak of 7.46 ppm for E_1_, indicating that the chemical environment of the Si atom has been changed (Figure 1c). It was proven that the Si atom could interact with heteroatoms (e.g., N or S) by accepting a lone pair of *p* electrons, resulting in S→Si or N→Si coordination bonds [44]. Thus, the present variation is apparently due to the formation of S→Si coordination.

### 2.3. Fluorescent Properties

**Figure 2:**
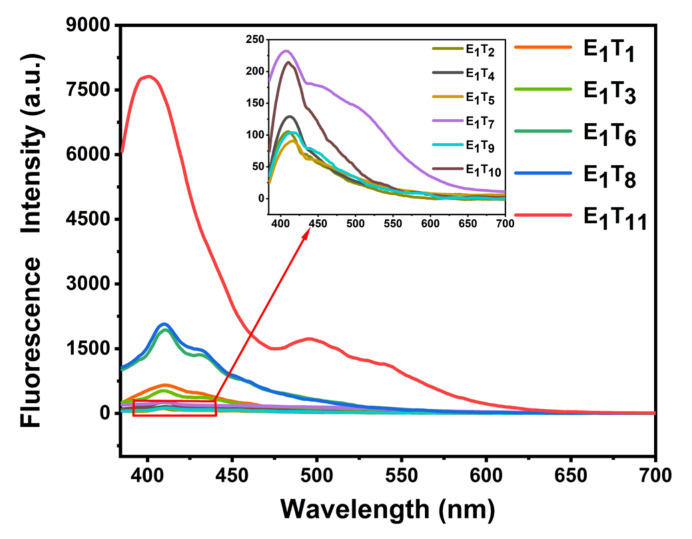
Fluorescence emission spectra of functionalized disiloxanes E_1_T_1_ to E_1_T_11_ in ethanol solutions excited at 365 nm (0.01 mol L^−1^).

**Figure 3:**
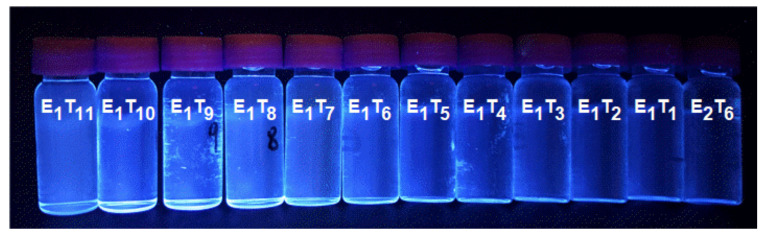
The photographs of E_1_T_1_ to E_1_T_11_ and E_2_T_6_ in ethanol solutions under 365 nm UV light.

Previous reports have demonstrated that the existence of heteroatom in organosilicon molecules or polymers could exhibit interesting nonconventional fluorescence [44]. The present sulfur-containing functionalized disiloxanes expectedly show nonconventional fluorescence. All the samples in ethanol (EtOH) solutions emit similar and strong blue fluorescence with the maximum emission wavelength (λ_max_) at ca. 410 nm when excited by 365 nm (Figure 2). Moreover, this phenomenon can be visualized under 365 nm ultraviolet (UV) light (Figure 3). Similar to previous reports [44], this nonconventional fluorescence was generated by through-space charge transfer from lone pairs of electrons in S atoms to the 3d empty orbital of Si atoms and aggregation of chromophores, including ester groups in E_1_T_6_ and aromatic units in E_1_T_7_ to E_1_T_11_. It is worth noting that E_1_T_11_ has the highest fluorescent intensities. Previous reports demonstrated that benzothiazole and its derivatives have excellent fluorescence due to the large delocalized π bond and rigid planar construction. Hence, the aromatic π–π conjugation contributes to the blue emission region with the λ_max_ at ca. 400 nm, in addition to the contribution from S→Si and N→Si coordination [45]. It is worth noting that the coordination is not only contributed by the thioether group but also by the S and N from thiazole units. These combined contributions lead to the highest fluorescence intensity. In addition, a weak emission peak at 497 nm was found. This finding is probably due to higher electronic conjugation induced by the π–π interaction between aromatic thiazole groups [46], while S→Si and N→Si coordination facilitates this interaction. The combined contribution can also explain higher fluorescence intensities found in E_1_T_6_ and E_1_T_9_ than in other samples, because additional aggregation of ester groups and N→Si coordination contributed by imidazole groups [24,47,48], exist within E_1_T_6_ and E_1_T_9_, respectively.

**Figure 4:**
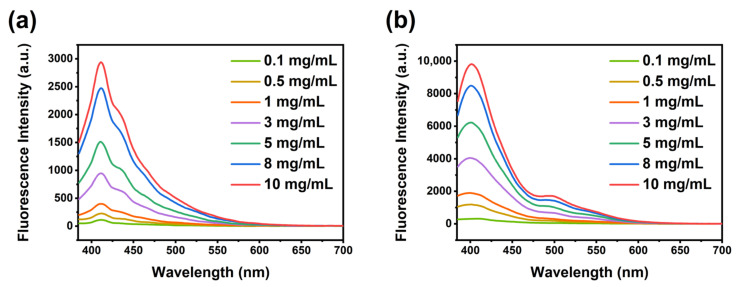
(**a**) Fluorescence emission spectra of E_1_T_6_ with different concentrations excited at 365 nm. (**b**) Fluorescence emission spectra of E_1_T_11_ with different concentrations excited at 365 nm.

The nonconventional fluorescent properties of these polysiloxanes can also be determined by the fluorescence variation in different concentrations in solutions. Figure 4 shows the fluorescence spectra of E_1_T_6_ and E_1_T_11_ in ethanol at different concentrations as the examples (see Figures S20–S22 for other samples). Their fluorescence intensities were progressively enhanced with increasing concentrations. This phenomenon is a typical example of aggregation-induced emission enhancement (AIEE), which is apparently induced by the aggregation of nonconventional chromophores, including X→Si (X = S or N) coordination bonds in these compounds and ester groups in E_1_T_6_ [47,49]. In addition, a new peak at ca. 500 nm could be observed at a high concentration of E_1_T_11_, which could be assigned to the extended conjugation formed by the aggregation of the thiazole groups.

**Figure 5:**
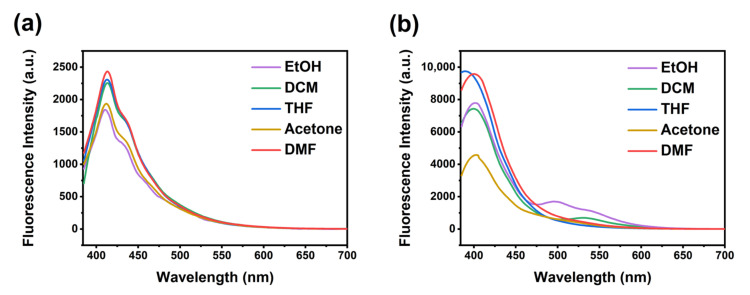
(**a**) Fluorescence emission spectra of E_1_T_6_ in different solvents excited at 365 nm (0.01 mol/L). (**b**) Fluorescence emission spectra of E_1_T_11_ in different solvents excited at 365 nm (0.01 mol/L).

Generally, the fluorescence property depends on the environment. Therefore, the photophysical characteristics of these sulfur-containing functionalized disiloxanes were investigated in various organic solvents. Figure 5 shows the fluorescence spectra of E_1_T_6_ and E_1_T_11_ in different solvents excited at 365 nm (Figures S23–S25 for other samples). For E_1_T_6_, the emission regions were similar in different solvents, but their fluorescence intensity varied. The fluorescence emission intensity became weaker in the order of dimethylformamide (DMF), THF, dichloromethane (DCM), ethanol, and acetone. The same variation trend of emission intensity was also found in E_1_T_11_ (Figure 5b). This finding may be due to the participation of the electron-rich solvents (lone pairs) in the aggregation of nonconventional chromophores and consequently physically altered the extended electronic conjugation. In addition, the polarity of the solvents can also influence the fluorescence. As a result, these factors affect the emission behavior [50].

**Scheme 3:**
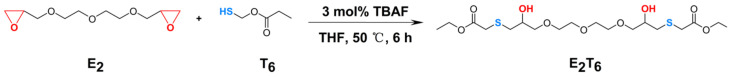
Synthesis route of the model compound E_2_T_6_.

To further prove the contribution of S→Si or Si–O to the nonconventional fluorescence, a model compound E_2_T_6_, which has similar functional groups with E_1_T_6_ but without Si–O–Si linker, was synthesized by the thiol–epoxy reaction of diethylene glycol diglycidyl ether (E_2_) and ethyl thioglycolate (T_6_) (Scheme 3).

**Figure 6:**
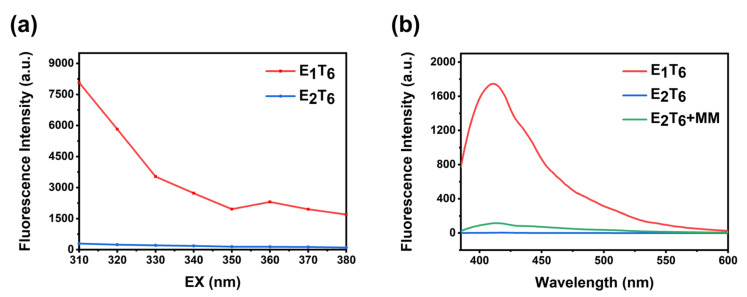
(**a**) The relative fluorescence intensity of E_2_T_6_ and E_1_T_6_ in THF solution (0.01 mol L^−1^). (**b**) Fluorescence emission spectra of E_1_T_6_, E_2_T_6,_ and E_2_T_6_/MM mixture in THF solution excited at 365 nm (MM was insoluble in ethanol at room temperature, 0.01 mol L^−1^).

As illustrated in Table 3, although E_2_T_6_ can also emit blue fluorescence and have similar emission wavelengths to E_1_T_6_, its fluorescence intensity is much lower than that of E_1_T_6_ under the same excitation wavelength (Figure 6a), indicating the important role of Si–O–Si in the enhancement of fluorescence intensity. This Si-O-Si induced enhancement can be found even without direct introduction in the structures. As shown in Figure 6b, the fluorescence intensity of E_2_T_6_ was obviously enhanced after adding equimolar hexamethyldisiloxane (MM), although it was still lower than that of E_1_T_6_. These results indicate that the interaction of S→Si coordination bonds can increase the aggregation of ester groups and thus enhance the fluorescence intensity.

## 3. Materials and Methods

### 3.1. Materials

All reagents and solvents were obtained from commercial suppliers and used without further purification unless otherwise noted. 1,3-bis(3-glycidoxypropyl)tetramethy-ldisiloxane (≥98%) was supplied by Hangzhou Sloan Materials Technology Co., Ltd. (Hangzhou, China). Diethylene glycol diglycidyl ether (68%) was purchased from Jinan Jing Shui Biotechnology Co., Ltd. (Jinan, China). Tetrabutylammonium fluoride (TBAF), lithium hydroxide (LiOH), 1-dodecanethiol (≥98%), 2-mercaptoethanol (≥99%), 1-butanethiol (≥97%), 2-propenthiol (50%), 3-methyl-2-butanethiol (≥98%), ethyl thioglycolate (≥98%), furfuryl mercaptan (≥98%), methimazole (≥98%), 3-methylbenzenethiol (≥97%), benzyl mercaptan (≥98%), and 2-mercaptobenzothiazole (≥98%) were purchased from MACKLIN Chemical Reagent Co., Ltd. (Shanghai, China). Tetrahydrofuran (THF), dichloromethane (DCM), ethyl acetate (EA), petroleum ether (PE), acetone, ethanol, and dimethylformamide (DMF) all with Grade AR were purchased from Tianjin Fuyu Chemical Reagent Co., Ltd. (Tianjin, China).

### 3.2. Methods

Fourier transform infrared (FT-IR) spectra were conducted on a Bruker TENSOR-27 FT-IR spectrophotometer (Germany) using the KBr pellet technique and recorded in the range of 400 cm^−1^ to 4000 cm^−1^. ^1^H NMR, ^13^C NMR, and ^29^Si NMR were recorded on a Bruker AVANCE 400 NMR spectrometer (Rheinstetten, Germany) operating at 25 °C using CDCl_3_ as solvent and without tetramethylsilane as an interior label, and using Software MestReNova-14.0.0-23239 to process the data. High-resolution mass spectra (HR-MS) were recorded on UPLC–QTOF–MS (Bruker, Bremen, Germany) with ESI^+^, source spray voltage 2.6 kV and capillary temperature 180.00 °C, and data processing was performed using Bruker Compass Data Analysis 4.4.2. The fluorescent emission spectra of the samples were determined at room temperature with a F-7000 fluorescence spectrophotometer (Hitachi, Tokyo, Japan) using a monochromated Xe lamp as an excitation source. The contents of C, H, N, and S were characterized by the Elementar Vario EL III elemental analyzer (Munich, Germany).

### 3.3. General Procedure for the Synthesis of Sulfur-Containing Functionalized Disiloxanes E_1_T_1_ to E_1_T_11_

1,3-bis(3-glycidoxypropyl)-1,1,3,3-tetramethyldisiloxane (E_1_, 362.6 mg, 1 mmol), thiol compounds (T_n_, 2.5 mmol), and tetrahydrofuran (THF, 0.6 mL) were placed into a 25 mL round-bottom flask with a magnetic stirrer. Then, tetrabutylammonium fluoride (TBAF, 7.9 mg, 0.03 mmol) was added to the mixture at 0 °C. After removing the cooling, the mixture was heated at 50 °C for 6 h under stirring. Then, the mixture was cooled to room temperature and dichloromethane DCM (100 mL) was added. The mixture was washed with water and the organic layer was separated and dried over anhydrous magnesium sulfate. After the filtration, the solvent was removed under reduced pressure to yield the crude product. The pure products E_1_T_1_ to E_1_T_11_ were then purified by column chromatography on silica gel using PE/EA as an eluent.

*1,3-bis(3-(dodecylthiopropoxy)-2-ol-propyl)-1,1,3,3-tetramethyldisiloxane (E_1_T_1_)*: The synthesis was performed according to general procedure using 1-dodecanethiol (T_1_, 506.0 mg) as a thiol compound. E_1_T_1_ was afforded as a colorless viscous oil (736.0 mg, yield: 96.0%). FT-IR (KBr pellet cm^−1^): ν = 3460, 2928, 2858, 1464, 1255, 1119, 1065, 843, 795. ^1^H NMR (400 MHz, CDCl_3_): [δ, ppm] = 0.00 (s, 12H, −Si*CH_3_*), 0.39–0.48 (t, 4H, −Si*CH_2_*), 0.83 (m, 6H, −CH_2_*CH_3_*), 1.20–1.32 (m, 36H, −*(CH_2_)_9_*CH_3_), 1.53 (m, 8H, −SiCH_2_*CH_2_*, −SCH_2_*CH_2_*), 2.44–2.71 (m, 10H, −CH(*OH*)*CH_2_*S*CH_2_*), 3.31–3.51 (m, 8H, −*CH_2_*O*CH_2_*), 3.81 (m, 2H, −*CH*(OH)). ^13^C NMR (101 MHz, CDCl_3_): [δ, ppm] = 0.00, 13.83, 13.91, 22.38, 23.09, 28.49–29.47, 31.61, 32.30, 35.50, 68.85, 73.08, 73.95. ^29^Si NMR (79 MHz, CDCl_3_): [δ, ppm] = 7.70. HR-MS (FAB) calcd for C_40_H_86_O_5_S_2_Si_2_ (MH^+^): 767.5533; Found: 767.5535. Anal. Calcd for C_40_H_86_O_5_S_2_Si_2_ (%): C, 62.61; H, 11.30; S, 8.36. Found: C, 61.69; H, 10.18; S, 9.52.

*1,3-bis(3-(butylthiopropoxy)-2-ol-propyl)-1,1,3,3-tetramethyldisiloxane (E_1_T_2_)*: The synthesis was performed according to general procedure using 1-butanethiol (T_2_, 225.5 mg) as a thiol compound. E_1_T_2_ was obtained as a colorless viscous oil (518.0 mg). Yield: 95.5%. FT-IR (KBr pellet cm^−1^): ν = 3456, 2949, 2868, 1464, 1418, 1259, 1188, 1121, 1063, 845, 795. ^1^H NMR (400 MHz, CDCl_3_): [δ, ppm] = 0.00 (s, 12H, −Si*CH_3_*), 0.40–0.49(t, 4H, −Si*CH_2_*), 0.86 (t, 6H, −CH_2_*CH_3_*), 1.28–1.42 (m, 4H, −*CH_2_*CH_3_), 1.46–1.60 (m, 8H, −SiCH_2_*CH_2_*, −SCH_2_*CH_2_*), 2.45–2.68 (m, 8H, −*CH_2_*S*CH_2_*), 2.76 (s, 2H, −CH(*OH*)), 3.33–3.50 (m, 8H, −*CH_2_*O*CH_2_*), 3.77–3.87 (m, 2H, −*CH*(OH)). ^13^C NMR (101 MHz, CDCl_3_): [δ, ppm] = 0.11, 13.69, 16.44, 22.00, 24.63, 32.07, 32.20, 36.21, 70.16, 73.49, 74.09. ^29^Si NMR (79 MHz, CDCl_3_): [δ, ppm] = 7.73. HR-MS (FAB) calcd for C_24_H_54_O_5_S_2_Si_2_ (MH^+^): 543.3029; Found: 543.3031. Anal. Calcd for C_24_H_54_O_5_S_2_Si_2_ (%): C, 53.09; H, 10.02; S, 11.81. Found: C, 52.58; H, 10.03; S, 12.08.

*1,3-bis(3-((2-hydroxyethyl)-thiopropoxy)-2-ol-propyl)-1,1,3,3-tetramethyldisiloxane (E_1_T_3_)*: The synthesis was performed according to general procedure using 2-mercaptoethanol (T_3_, 195.0 mg) as a thiol compound. E_1_T_3_ was obtained as a colorless viscous oil (494.4 mg). Yield: 95.4%. FT-IR (KBr pellet cm^−1^): ν = 3410, 2941, 2869, 1651, 1410, 1256, 1182, 1115, 1057, 845, 791. ^1^H NMR (400 MHz, CDCl_3_): [δ, ppm] = 0.00 (s, 12H, −Si*CH_3_*), 0.39–0.48 (m, 4H, −Si*CH_2_*), 1.54 (dq, 4H, −SiCH_2_*CH_2_*), 2.52–2.74 (m, 8H, −*CH_2_*S*CH_2_*), 3.29–3.47 (m, 8H, −*CH_2_*O*CH_2_*), 3.56 (s, 4H, −CH_2_*OH*, −CH(*OH*)), 3.70 (t, 4H, −*CH_2_*OH), 3.86 (m, 2H, −*CH*(OH)). ^13^C NMR (101 MHz, CDCl_3_): [δ, ppm] = 0.00, 13.81, 22.98, 35.39, 35.41, 60.87, 69.49, 73.03, 73.90. ^29^Si NMR (79 MHz, CDCl_3_): [δ, ppm] = 7.70. HR-MS (FAB) calcd for C_20_H_46_O_7_S_2_Si_2_ (MH^+^): 519.2302; Found: 519.2305. Anal. Calcd for C_20_H_46_O_7_S_2_Si_2_ (%): C, 46.30; H, 8.94; S, 12.36. Found: C, 45.64; H, 8.93; S, 12.40.

*1,3-bis(3-(allylthiopropoxy)-2-ol-propyl)-1,1,3,3-tetramethyldisiloxane (E_1_T_4_)*: The synthesis was performed according to general procedure using 2-propenthiol (T_4_, 370.0 mg) as a thiol compound. E_1_T_4_ was obtained as a colorless viscous oil (480.1 mg). Yield: 94.2%. FT-IR (KBr pellet cm^−1^): ν = 3456, 2924, 2868, 1639, 1414, 1256, 1188, 1121, 1065, 835, 791. ^1^H NMR (400 MHz, CDCl_3_): [δ, ppm] = 0.00 (s, 12H, −Si*CH_3_*), 0.40–0.49 (m, 4H, −Si*CH_2_*), 1.48–1.60 (m, 4H, −SiCH_2_*CH_2_*), 2.44–2.65 (m, 4H, −CH(OH)*CH_2_*SCH_2_), 2.85 (s, 2H, −CH(*OH*)), 3.10 (dd, 4H, −CH(OH)CH_2_S*CH_2_*), 3.30–3.50 (m, 8H, −*CH_2_*O*CH_2_*), 3.82 (q, 2H, −*CH*(OH)), 4.99–5.16 (m, 4H, −CH=*CH_2_*), 5.66–5.81 (m, 2H, −*CH*=CH_2_). ^13^C NMR (101 MHz, CDCl_3_): [δ, ppm] = 0.00, 13.90, 23.07, 33.79, 34.78, 68.87, 73.07, 73.90, 117.11, 133.80. ^29^Si NMR (79 MHz, CDCl_3_): [δ, ppm] = 7.73. HR-MS (FAB) calcd for C_22_H_46_O_5_S_2_Si_2_ (MH^+^): 511.2403; Found: 511.2402. Anal. Calcd for C_22_H_46_O_5_S_2_Si_2_ (%): C, 51.72; H, 9.08; S, 12.55. Found: C, 51.27; H, 8.47; S, 13.05.

*1,3-bis(3-((3-methylbutan-2-methyl)-thiopropoxy)-2-ol-propyl)-1,1,3,3-tetramethyldisiloxane (E_1_T_5_)*: The synthesis was performed according to general procedure using 3-methyl-2-butanethiol (T_5_, 260.3 mg) as a thiol compound. E_1_T_5_ was obtained as a colorless viscous oil (514.4 mg). Yield: 90.2%. FT-IR (KBr pellet cm^−1^): ν = 3456, 2964, 2869, 1462, 1379, 1249, 1109, 1061, 843, 795. ^1^H NMR (400 MHz, CDCl_3_): [δ, ppm] = 0.00 (s, 12H, −Si*CH_3_*), 0.39–0.51 (m, 4H, −Si*CH_2_*), 0.91 (dd, 12H, −CH*(CH_3_)_2_*), 1.17 (d, 6H, −SCH(*CH_3_*)), 1.48–1.61 (m, 4H, −SiCH_2_*CH_2_*), 1.72–1.86 (m, 2H, −*CH*(CH_3_)_2_), 2.52–2.71 (m, 6H, −*CH_2_*S*CH*), 2.85 (m, 2H, −CH(*OH*)), 3.32–3.50 (m, 8H, −*CH_2_*O*CH_2_*), 3.80 (m, 2H, −*CH*(OH)). ^13^C NMR (101 MHz, CDCl_3_): [δ, ppm] = 0.01, 13.93, 17.35, 18.14, 19.55, 23.09, 32.58, 34.58, 47.22, 69.07, 73.08, 73.95. ^29^Si NMR (79 MHz, CDCl_3_): [δ, ppm] = 7.74. HR-MS (FAB) calcd for C_26_H_58_O_5_S_2_Si_2_ (MH^+^): 571.3342; Found: 571.3345. Anal. Calcd for C_26_H_58_O_5_S_2_Si_2_ (%): C, 54.69; H, 10.24; S, 11.23. Found: C, 52.85; H, 10.09; S, 9.99.

*1,3-bis(3-(ethyl-3-acetate-thiopropoxy)-2-ol-propyl)-1,1,3,3-tetramethyldisiloxane (E_1_T_6_)*: The synthesis was performed according to general procedure, ethyl thioglycolate (T_6_, 300.0 mg) as a thiol compound. E_1_T_6_ was obtained as a colorless viscous oil (572.1 mg). Yield: 95.0%. FT-IR (KBr pellet cm^−1^): ν = 3469, 2939, 2870, 1738, 1466, 1408, 1265, 1123, 1042, 843, 787. ^1^H NMR (400 MHz, CDCl_3_): [δ, ppm] = 0.00 (s, 12H, −Si*CH_3_*), 0.44 (m, 4H, −Si*CH_2_*), 1.23 (m, 6H, −CH_2_*CH_3_*), 1.53 (m, 4H, −SiCH_2_*CH_2_*), 2.62–2.83 (m, 4H, −S*CH_2_*CH(OH)), 2.88 (s, 2H, −CH(*OH*)), 3.25 (t, 4H, −S*CH_2_*C(O)), 3.31–3.51 (m, 8H, −*CH_2_*O*CH_2_*), 3.88 (m, 2H, −*CH*(OH)), 4.14 (m, 4H, −O*CH_2_*CH_3_). ^13^C NMR (101 MHz, CDCl_3_): [δ, ppm] = 0.00, 13.83, 13.88, 23.05, 33.94, 36.14, 61.18, 69.10, 72.94, 73.93, 170.47. ^29^Si NMR (79 MHz, CDCl_3_): [δ, ppm] = 7.65. HR-MS (FAB) calcd for C_24_H_50_O_9_S_2_Si_2_ (MH^+^): 603.2513; Found: 603.2520. Anal. Calcd for C_24_H_50_O_9_S_2_Si_2_ (%): C, 47.81; H, 8.36; S, 10.63. Found: C, 46.86; H, 8.40; S, 10.68.

*1,3-bis(3-((furanyl-methyl)-thiopropoxy)-2-ol-propyl)-1,1,3,3-tetramethyldisiloxane (E_1_T_7_)*: The synthesis was performed according to general procedure using furfuryl mercaptan (T_7_, 285.0 mg) as a thiol compound. E_1_T_7_ was obtained as a pale-yellow viscous oil (559.0 mg). Yield: 94.7%. FT-IR (KBr pellet cm^−1^): ν = 3452, 3119, 2930, 2870, 1506, 1414, 1254, 1119, 1065, 868, 796. ^1^H NMR (400 MHz, CDCl_3_): [δ, ppm] = 0.01 (s, 12H, −Si*CH_3_*), 0.39–0.49 (m, 4H, −Si*CH_2_*), 1.47–1.60 (m, 4H, −SiCH_2_*CH_2_*), 2.50–2.68 (m, 4H, −S*CH_2_*CH(OH)), 2.81 (t, 2H, −CH(*OH*)), 3.31–3.48 (m, 8H, −*CH_2_*O*CH_2_*), 3.68–3.74 (m, 4H, −CHCH_2_S*CH_2_*), 3.75–3.84 (m, 2H, −*CH*(OH)), 6.11–6.27 (m, 4H, −OCH=*CHCH*=C, Furan ring), 7.27–7.33 (m, 2H, −O*CH*=CHCH=C, Furan ring). ^13^C NMR (101 MHz, CDCl_3_): [δ, ppm] = 0.00, 13.88, 23.05, 28.37, 34.71, 69.03, 72.97, 73.87, 107.41, 110.10, 141.86, 151.05. ^29^Si NMR (79 MHz, CDCl_3_): [δ, ppm] = 7.74. HR-MS (FAB) calcd for C_26_H_46_O_7_S_2_Si_2_ (MH^+^): 591.2297; Found: 591.2292. Anal. Calcd for C_26_H_46_O_7_S_2_Si_2_ (%): C, 52.85; H, 7.85; S, 10.85. Found: C, 51.16; H, 7.23; S, 10.13.

*1,3-bis(3-((1-methylimidazol)-thiopropoxy)-2-ol-propyl)-1,1,3,3-tetramethyldisiloxane (E_1_T_8_)*: The synthesis was performed according to general procedure, methimazole (T_8_, 285.0 mg) as a thiol compound. E_1_T_8_ was obtained as a pale-yellow viscous oil (545.0 mg). Yield: 92.3%. FT-IR (KBr pellet cm^−1^): ν = 3390, 3198, 3117, 2937, 2865, 1674, 1514, 1466, 1258, 1117, 1055. ^1^H NMR (400 MHz, CDCl_3_): [δ, ppm] = 0.00 (s, 12H, −Si*CH_3_*), 0.39–0.49 (m, 4H, −Si*CH_2_*), 1.46–1.59 (m, 4H, −SiCH_2_*CH_2_*), 3.05–3.27 (m, 4H, −S*CH_2_*CH(OH)), 3.34–3.52 (m, 8H, −*CH_2_*O*CH_2_*), 3.54 (s, 6H, −Imidazole−*CH_3_*), 4.05 (m, 2H, −*CH*(OH)), 5.43–6.04 (s, 2H, -CH(*OH*)), 6.85 (m, 4H, −*Imidazole*). ^13^C NMR (101 MHz, CDCl_3_): [δ, ppm] = 0.00, 13.90, 23.08, 33.16, 37.25, 70.25, 72.89, 73.86, 121.83, 127.73, 142.69. ^29^Si NMR (79 MHz, CDCl_3_): [δ, ppm] = 7.73. HR-MS (FAB) calcd for C_24_H_46_N_4_O_5_S_2_Si_2_ (MH^+^): 591.2526; Found: 591.2534. Anal. Calcd for C_24_H_46_N_4_O_5_S_2_Si_2_ (%): C, 48.78; H, 7.85; N, 9.48; S, 10.85. Found: C, 47.94; H, 8.07; N, 10.43; S, 10.39.

*1,3-bis(3-(m-tolyl-thiopropoxy)-2-ol-propyl)-1,1,3,3-tetramethyldisiloxane (E_1_T_9_)*: The synthesis was performed according to general procedure using 3-methylbenzenethiol (T_9_, 310.0 mg) as a thiol compound. E_1_T_9_ was obtained as a colorless viscous oil (589.0 mg). Yield: 96.5%. FT-IR (KBr pellet cm^−1^): ν = 3454, 3051, 2939, 2864, 1587, 1475, 1412, 1258, 1180, 1111, 1053. ^1^H NMR (400 MHz, CDCl_3_): [δ, ppm] = 0.00 (s, 12H, −Si*CH_3_*), 0.39–0.50 (m, 4H, −Si*CH_2_*), 1.46–1.58 (m, 4H, −SiCH_2_*CH_2_*), 2.24 (s, 6H, −Phenyl−*CH_3_*), 2.91–3.08 (m, 6H, −S*CH_2_*CH(*OH*)), 3.32–3.48 (m, 8H, −*CH_2_*O*CH_2_*), 3.82 (m, 2H, −*CH*(OH)), 6.91–7.15 (m, 8H, −*Phenyl*). ^13^C NMR (101 MHz, CDCl_3_): [δ, ppm] = 0.00, 13.82, 20.93, 22.99, 36.85, 68.68, 72.62, 73.76, 125.96, 126.64, 128.39, 129.56, 135.27, 138.18. ^29^Si NMR (79 MHz, CDCl_3_): [δ, ppm] = 7.74. HR-MS (FAB) calcd for C_30_H_50_O_5_S_2_Si_2_ (MH^+^): 611.2716; Found: 611.2725. Anal. Calcd for C_30_H_50_O_5_S_2_Si_2_ (%): C, 58.97; H, 8.25; S, 10.49. Found: C, 58.38; H, 8.31; S, 10.49.

*1,3-bis(3-(benzyl-thiopropoxy)-2-ol-propyl)-1,1,3,3-tetramethyldisiloxane (E_1_T_10_)*: The synthesis was performed according to general procedure using, benzyl mercaptan (T_10_, 310.0 mg) as a thiol compound. E_1_T_10_ was obtained as a colorless viscous oil (572.4 mg). Yield: 93.8%. FT-IR (KBr pellet cm^−1^): ν = 3447, 3022, 2928, 2868, 1603, 1495, 1416, 1258, 1123, 1049, 706. ^1^H NMR (400 MHz, CDCl_3_): [δ, ppm] = 0.00 (s, 12H, −Si*CH_3_*), 0.38–0.48 (m, 4H, −Si*CH_2_*), 1.46–1.58 (m, 4H, −SiCH_2_*CH_2_*), 2.50 (m, 4H, −S*CH_2_*CH(OH)), 2.68–2.74 (m, 2H, −CH(*OH*)), 3.30–3.44 (m, 8H, −*CH_2_*O*CH_2_*), 3.68 (s, 4H, -CHCH_2_S*CH_2_*), 3.79 (m, 2H, −*CH*(OH)), 7.14–7.28 (m, 10H, −*Phenyl*). ^13^C NMR (101 MHz, CDCl_3_): [δ, ppm] = 0.00, 13.84, 23.01, 34.36, 36.19, 68.84, 72.99, 73.80, 126.68, 128.13, 128.55, 137.79. ^29^Si NMR (79 MHz, CDCl_3_): [δ, ppm] = 7.78. HR-MS (FAB) calcd for C_30_H_50_O_5_S_2_Si_2_ (MH^+^): 611.2716; Found: 611.2729. Anal. Calcd for C_30_H_50_O_5_S_2_Si_2_ (%): C, 58.97; H, 8.25; S, 10.49. Found: C, 57.95; H, 8.32; S, 10.65.

*1,3-bis(3-(benzothiazolyl-thiopropoxy)-2-ol-propyl)-1,1,3,3-tetramethyldisiloxane (E_1_T_11_)*: The synthesis was performed according to general procedure using 2-mercaptobenzothiazole (T_11_, 417.5 mg) as a thiol compound. E_1_T_11_ was obtained as a yellow viscous oil (622.3 mg). Yield: 89.4%. FT-IR (KBr pellet cm^−1^): ν = 3418, 3068, 2939, 2872, 1568, 1462, 1425, 1256, 1186, 1065, 845. ^1^H NMR (400 MHz, CDCl_3_): [δ, ppm] = 0.00 (s, 12H, −Si*CH_3_*), 0.40–0.50 (m, 4H, −Si*CH_2_*), 1.54 (dq, 4H, −SiCH_2_*CH_2_*), 3.34–3.55 (dt, 12H, −*CH_2_*O*CH_2_*CH(OH)*CH_2_*), 4.14 (m, 2H, −*CH*(OH)), 4.64 (d, 2H, −CH(*OH*)), 7.15–7.69 (m, 8H, −*Phenyl*). ^13^C NMR (101 MHz, CDCl_3_): [δ, ppm] = 0.00, 13.86, 23.05, 37.04, 69.54, 72.68, 73.88, 120.62, 120.87, 124.09, 125.78, 134.96, 152.10, 167.45. ^29^Si NMR (79 MHz, CDCl_3_): [δ, ppm] = 7.80. HR-MS (FAB) calcd for C_30_H_44_N_2_O_5_S_4_Si_2_ (MH^+^): 697.1750; Found: 697.1760. Anal. Calcd for C_30_H_44_N_2_O_5_S_4_Si_2_ (%): C, 51.69; H, 6.36; N, 4.02; S, 18.40. Found: C, 49.95; H, 6.39; N, 4.99; S, 17.65.

### 3.4. Synthesis of bis(2-(ethyl-3-acetate-thiopropoxy)-2-ol-ethyl)-ether (E_2_T_6_)

The synthesis of E_2_T_6_ was performed according to general procedure based on diethylene glycol diglycidyl ether (E_2_, 218.1 mg, l mmol) and ethyl thioglycolate (T_6_, 300.0 mg, 2.5 mmol). E_2_T_6_ was obtained as a colorless viscous oil (435.3 mg) with the same synthesis procedure of E_1_T_1_. Yield: 95.0%. FT-IR (KBr pellet cm^−1^): ν = 3446.67, 2912.41, 2881, 1732.01, 1467.78, 1284.55, 1132.17, 1031.88. ^1^H NMR (400 MHz, CDCl_3_): [δ, ppm] = 1.23 (t, 6H, −CH_2_*CH_3_*), 2.71 (m, 4H, −S*CH_2_*CH(OH)), 3.25 (d, 4H, −S*CH_2_*COO), 3.43–3.66 (m, 14H, −O*CH_2_CH_2_*O*CH_2_*CH(*OH*)), 3.85–3.93 (m, 2H, −*CH*(OH)), 4.14 (m, 4H, −*CH_2_*CH_3_). ^13^C NMR (101 MHz, CDCl_3_): [δ, ppm] = 14.15, 34.30, 35.75, 61.46, 69.50, 70.41, 70.53, 74.28, 170.71.

## 4. Conclusions

In summary, we developed an efficient synthetic strategy, i.e., a thiol–epoxy reaction, for the synthesis of novel sulfur-containing functionalized disiloxanes from 1,3-bis(3-glycidoxypropyl)-1,1,3,3-tetramethyldisiloxane and different thiol compounds. The study found that both lithium hydroxide (LiOH) and tetrabutylammonium fluoride (TBAF) have high catalytic activity, and TBAF in THF is universal for the synthesis of these compounds because THF can dissolve most thiol compounds. The reactions can be conducted under mild reaction conditions with high reaction yields and regioselectivity, no by-products, and good functional group tolerance. Furthermore, these new disiloxanes exhibit typical aggregation-induced emission enhancement (AIEE) characteristics and can emit strong blue fluorescence under the action of the unconventional fluorescent chromophores S→Si or N→Si coordination bonds. In addition, the S→Si coordination bonds can also increase the aggregation of the nonconventional fluorescent clusters and thus enhance the fluorescence intensity. These results demonstrate that the thiol–epoxy reaction could be developed as an efficient organosilicon synthetic methodology for constructing various organosilicon compounds or polymers with new structures and functionalities from the low-cost epoxy-containing organosilicon raw materials. It is worth noting that although TBAF is an efficient catalyst for the synthesis of functionalized disiloxanes, it may be not adaptable for the synthesis of other organosilicon compounds, especially silsesquioxanes, because fluoride ions could react rapidly with silicon atoms and it may be a cause of complicated and unpredictable synthetic results. Further synthesis of other organosilicon materials and the reaction methods may be also optimized. Our group is currently utilizing this reaction to construct various novel organosilicon polymers and materials and find their applications in emerging fields, such as sensing and catalysis.

## Figures and Tables

**Table 1:** Optimization of the reaction condition of the thiol–epoxy reaction based on E_1_ and T_1_.

Entry *^a^*	Catalyst	Cat. Loading (mol%)	Time (h)	Temp (°C)	Conversion (%) *^c^*
1	LiOH	5	6	25	63.1
2	LiOH	5	12	25	99.5
3	LiOH	3	12	25	80.3
4	LiOH	1	12	25	39.8
5	TBAF *^b^*	8	12	25	81.3
6	TBAF *^b^*	8	12	50	99.5
7	TBAF *^b^*	5	12	50	99.4
8	TBAF *^b^*	3	12	50	98.6
9	TBAF *^b^*	1	12	50	92.3
10	TBAF *^b^*	0.8	12	50	80.0
11	TBAF *^b^*	3	6	50	96.7
12	TBAF *^b^*	3	3	50	91.8

*^a^* Reaction conditions: E_1_ (1 mmol), T_1_ (1.25 mmol), and catalyst. The amount of catalyst was calculated based on epoxy group. *^b^* The reactions were carried out in THF. *^c^* Determined by ^1^H NMR.

**Table 2:** The performance of optimized conditions in the thiol–epoxy reaction of E_1_ and various thiol compounds (T_n_).

Entry *^a^*	Substrates		Products		Time (h)	Conversion (%) *^b^*	Yields (%) *^c^*
1	T_1_	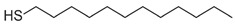	E_1_T_1_	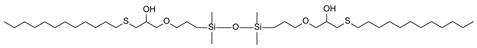	6	96.0	96.0
2	T_2_	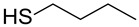	E_1_T_2_	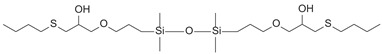	6	99.9	95.5
3	T_3_	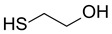	E_1_T_3_	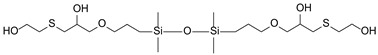	6	99.9	95.4
4	T_4_	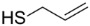	E_1_T_4_	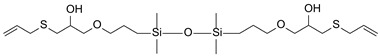	6	99.9	94.3
5	T_5_	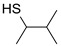	E_1_T_5_	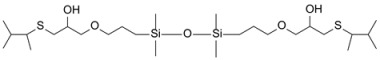	6	99.0	90.3
6	T_6_	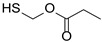	E_1_T_6_	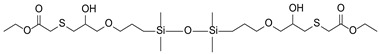	6	98.0	95.0
7	T_7_	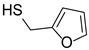	E_1_T_7_	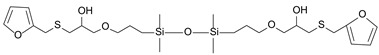	6	99.9	94.7
8	T_8_	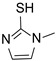	E_1_T_8_	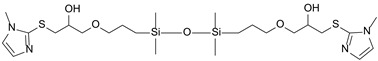	6	99.9	92.3
9	T_9_	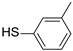	E_1_T_9_	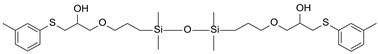	6	99.9	96.5
10	T_10_	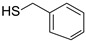	E_1_T_10_	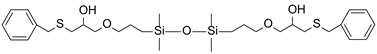	6	99.9	93.8
11	T_11_	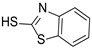	E_1_T_11_	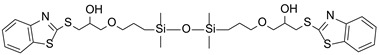	6	98.0	89.3
12	T_2_		E_1_T_2_		3	99.9	95.5
13	T_3_		E_1_T_3_		5	99.9	95.3
14	T_4_		E_1_T_4_		5	99.9	93.8
15	T_8_		E_1_T_8_		5	99.9	92.1
16	T_9_		E_1_T_9_		3	99.9	96.4

*^a^* Reaction conditions: E_1_ (1 mmol), substrates (1.25 mmol), TBAF (3 mol%), THF (0.6 mL), 50 °C, 6 h. *^b^* The conversion rate of epoxy groups determined by ^1^H NMR. *^c^* Isolated yields and confirmed by FT-IR, NMR, HR-MS, and elemental analysis.

**Table 3:** The excitation and emission of E_2_T_6_ and E_1_T_6_.

Entry	E_x_/nm	E_2_T_6_		E_1_T_6_	
		E_m_/nm	Intensity	E_m_/nm	Intensity
1	310	345	299.1	349	8069
2	320	357	245	363	5824
3	330	370	212.3	366	3534
4	340	381	184.8	386	2730
5	350	392	147.9	410	1959
6	360	404	139.7	409	2310
7	370	417	131.8	409	1951
8	380	430	102	412	1700

## Data Availability

Not applicable.

## Supplementary Materials

The following supporting information can be downloaded at: https://www.mdpi.com/article/10.3390/ijms24097785/s1.
