# Supplementary material for: Facile Synthesis of Sulfur-Containing Functionalized Disiloxanes with Nonconventional Fluorescence by Thiol–Epoxy Click Reaction
Source: Int J Mol Sci. 2023 Apr 24;24(9):7785. doi: 10.3390/ijms24097785 (PMC10177946; doi:10.3390/ijms24097785)
Supplement: Supplementary file 1 [file ijms-24-07785-s001.zip › ijms-2339988-supplementary.pdf]

Supporting Information

Facile Synthesis of Sulfur-Containing Functionalized Disiloxanes with Nonconventional Fluorescence by Thiol–Epoxy Click Reaction

Jing Tang [1,2], Shengyu Feng [2] and Dengxu Wang [1,2,*]

[1] Institute of Novel Semiconductors, State Key Laboratory of Crystal Materials, Shandong University, Jinan 250100, China

[2] National Engineering Research Center for Colloidal Materials & Key Laboratory of Special Functional Aggregated Materials, Ministry of Education, Shandong Key Laboratory of Advanced Organosilicon Materials and Technologies, School of Chemistry and Chemical Engineering, Shandong University, Jinan 250100, China

* Correspondence: dxwang@sdu.edu.cn

Contents

Figure S1. ^{29}Si NMR spectra of the reaction mixture after 6 h (TBAF, 10mol%)

Figure S2. (a) FT-IR spectra of functionalized disiloxane E_1T_1; (b) ^{1}H NMR spectrum of E_1T_1; (c) ^{29}Si NMR spectrum of E_1T_1; (d) ^{13}C NMR spectrum of E_1T_1.

Figure S3. (a) FT-IR spectra of functionalized disiloxane E_1T_2; (b) ^{1}H NMR spectrum of E_1T_2; (c) ^{29}Si NMR spectrum of E_1T_2; (d) ^{13}C NMR spectrum of E_1T_2.

Figure S4. (a) FT-IR spectra of functionalized disiloxane E_1T_3; (b) ^{1}H NMR spectrum of E_1T_3; (c) ^{29}Si NMR spectrum of E_1T_3; (d) ^{13}C NMR spectrum of E_1T_3.

Figure S5. (a) FT-IR spectra of functionalized disiloxane E_1T_4; (b) ^{1}H NMR spectrum of E_1T_4; (c) ^{29}Si NMR spectrum of E_1T_4; (d) ^{13}C NMR spectrum of E_1T_4.

Figure S6. (a) FT-IR spectra of functionalized disiloxane E_1T_5; (b) ^{1}H NMR spectrum of E_1T_5; (c) ^{29}Si NMR spectrum of E_1T_5; (d) ^{13}C NMR spectrum of E_1T_5.

Figure S7. (a) FT-IR spectra of functionalized disiloxane E_1T_6; (b) ^{1}H NMR spectrum of E_1T_6; (c) ^{29}Si NMR spectrum of E_1T_6; (d) ^{13}C NMR spectrum of E_1T_6.

Figure S8. (a) FT-IR spectra of functionalized disiloxane E_1T_7; (b) ^{1}H NMR spectrum of E_1T_7; (c) ^{29}Si NMR spectrum of E_1T_7; (d) ^{13}C NMR spectrum of E_1T_7.

Figure S9. (a) FT-IR spectra of functionalized disiloxane E_1T_8; (b) ^{1}H NMR spectrum of E_1T_8; (c) ^{29}Si NMR spectrum of E_1T_8; (d) ^{13}C NMR spectrum of E_1T_8.

Figure S10. (a) FT-IR spectra of functionalized disiloxane E_1T_9; (b) ^1H NMR spectrum of E_1T_9; (c) ^{29}Si NMR spectrum of E_1T_9; (d) ^{13}C NMR spectrum of E_1T_9.

Figure S11. (a) FT-IR spectra of functionalized disiloxane E_1T_{10}; (b) ^1H NMR spectrum of E_1T_{10}; (c) ^{29}Si NMR spectrum of E_1T_{10}; (d) ^{13}C NMR spectrum of E_1T_{10}.

Figure S12. (a) FT-IR spectra of functionalized disiloxane E_1T_{11}; (b) ^1H NMR spectrum of E_1T_{11}; (c) ^{29}Si NMR spectrum of E_1T_{11}; (d) ^{13}C NMR spectrum of E_1T_{11}.

Figure S13. (a) FT-IR spectra of functionalized disiloxane E_2T_6; (b) ^1H NMR spectrum of E_2T_6; (c) ^{13}C NMR spectrum of E_2T_6.

Figure S14. HR-MS spectra of functionalized disiloxane E_1T_1 and E_1T_2

Figure S15. HR-MS spectra of functionalized disiloxane E_1T_3 and E_1T_4

Figure S16. HR-MS spectra of functionalized disiloxane E_1T_5 and E_1T_6

Figure S17. HR-MS spectra of functionalized disiloxane E_1T_7 and E_1T_8

Figure S18. HR-MS spectra of functionalized disiloxane E_1T_9 and E_1T_{10}

Figure S19. HR-MS spectra of functionalized disiloxane E_1T_{11}

Figure S20. Fluorescence emission spectra of E_1T_1 (a), E_1T_2 (b), E_1T_3 (c) and E_1T_4 (d) with different concentration excited at 365 nm

Figure S21. Fluorescence emission spectra of E_1T_5 (a), E_1T_6 (b), E_1T_7 (c) and E_1T_8 (d) with different concentration excited at 365 nm

Figure S22. Fluorescence emission spectra of E_1T_9 (a), E_1T_{10} (b), and E_1T_{11} (c) with different concentration excited at 365 nm

Figure S23. Fluorescence emission spectra of E_1T_1 (a), E_1T_2 (b), E_1T_3 (c), and E_1T_4 (d) in different solvents excited at 365 nm

Figure S24. Fluorescence emission spectra of E_1T_5 (a), E_1T_6 (b), E_1T_7 (c), and E_1T_8 (d) in different solvents excited at 365 nm

Figure S25. Fluorescence emission spectra of E_1T_9 (a), E_1T_{10} (b), and E_1T_{11} (c) in different solvents excited at 365 nm

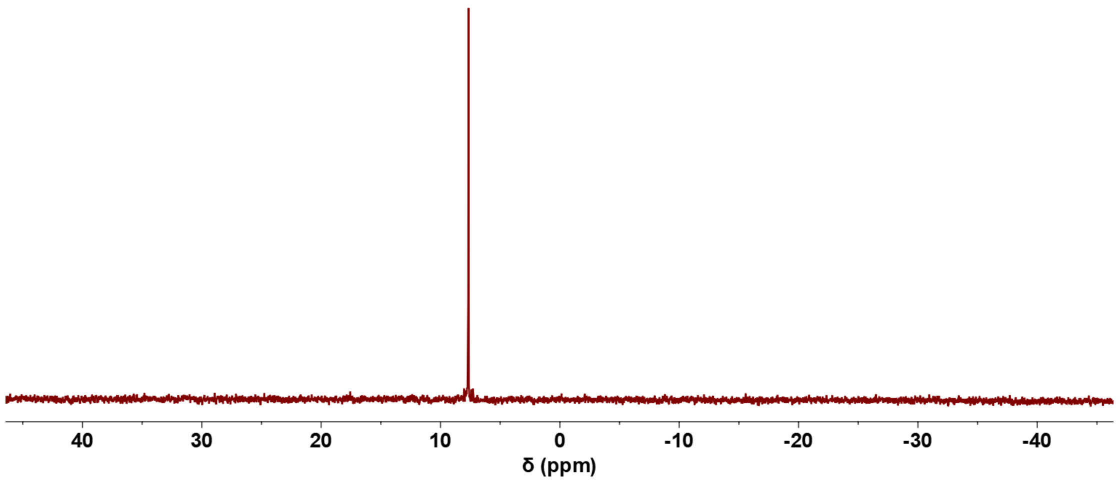

Figure S1. ^{29}Si NMR spectra of the reaction mixture after 6 h (TBAF, 10mol%)

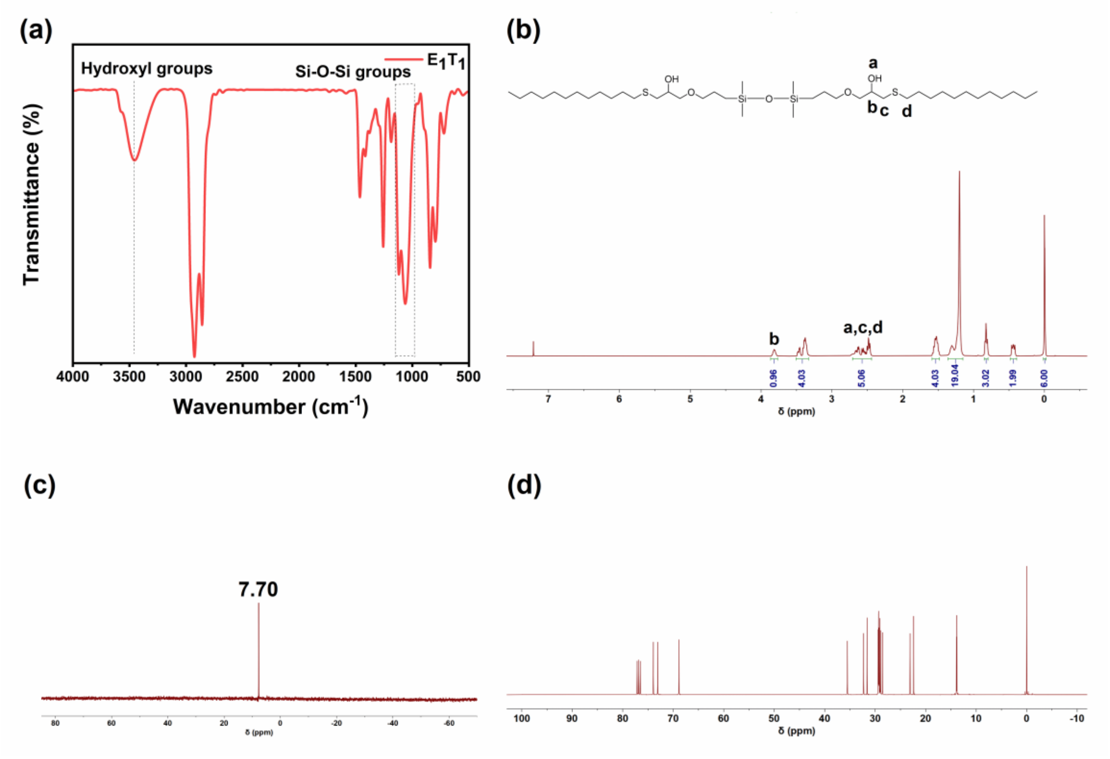

Figure S2. (a) FT-IR spectra of functionalized disiloxane E_1T_1; (b) ^{1}H NMR spectrum of E_1T_1; (c) ^{29}Si NMR spectrum of E_1T_1; (d) ^{13}C NMR spectrum of E_1T_1.

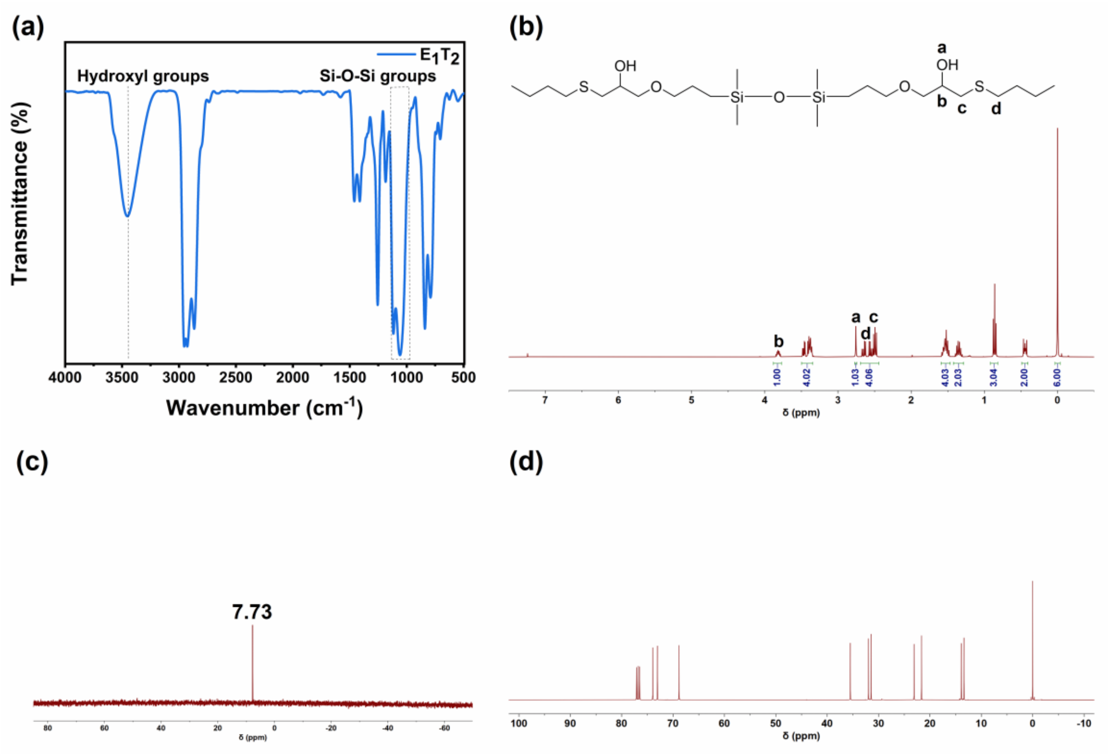

Figure S3. (a) FT-IR spectra of functionalized disiloxane E_1T_2; (b) ^{1}H NMR spectrum of E_1T_2; (c) ^{29}Si NMR spectrum of E_1T_2; (d) ^{13}C NMR spectrum of E_1T_2.

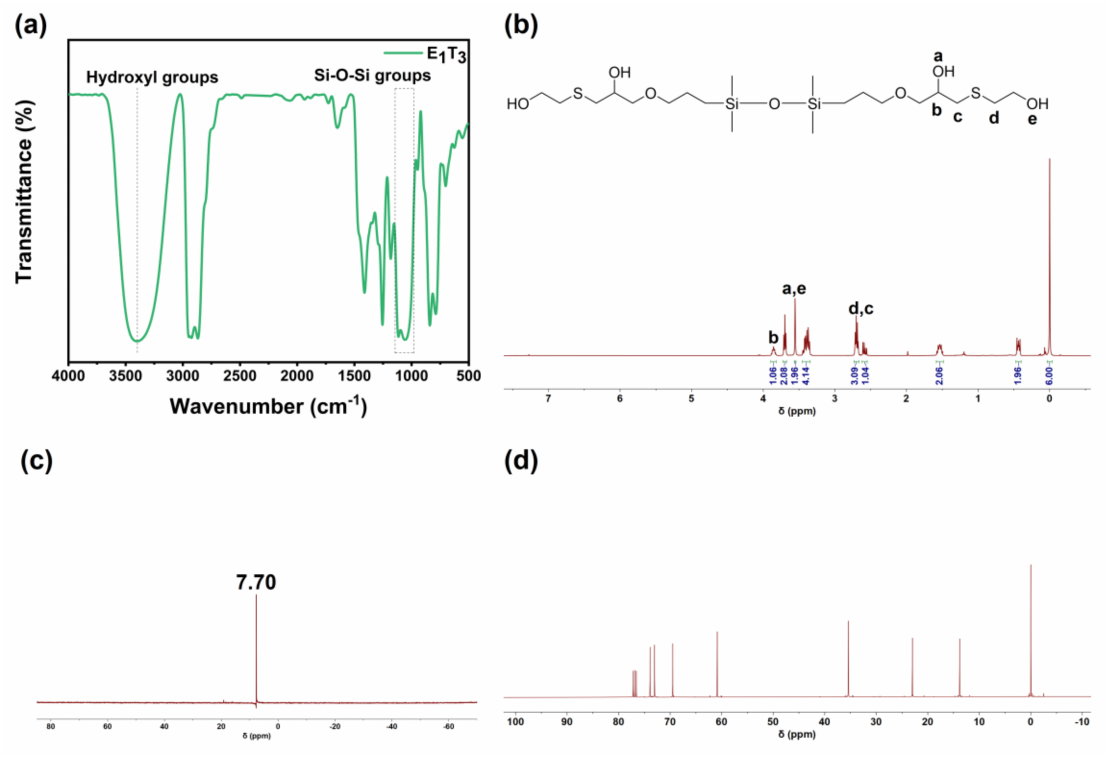

Figure S4. (a) FT-IR spectra of functionalized disiloxane E_1T_3; (b) ^{1}H NMR spectrum of E_1T_3; (c) ^{29}Si NMR spectrum of E_1T_3; (d) ^{13}C NMR spectrum of E_1T_3.

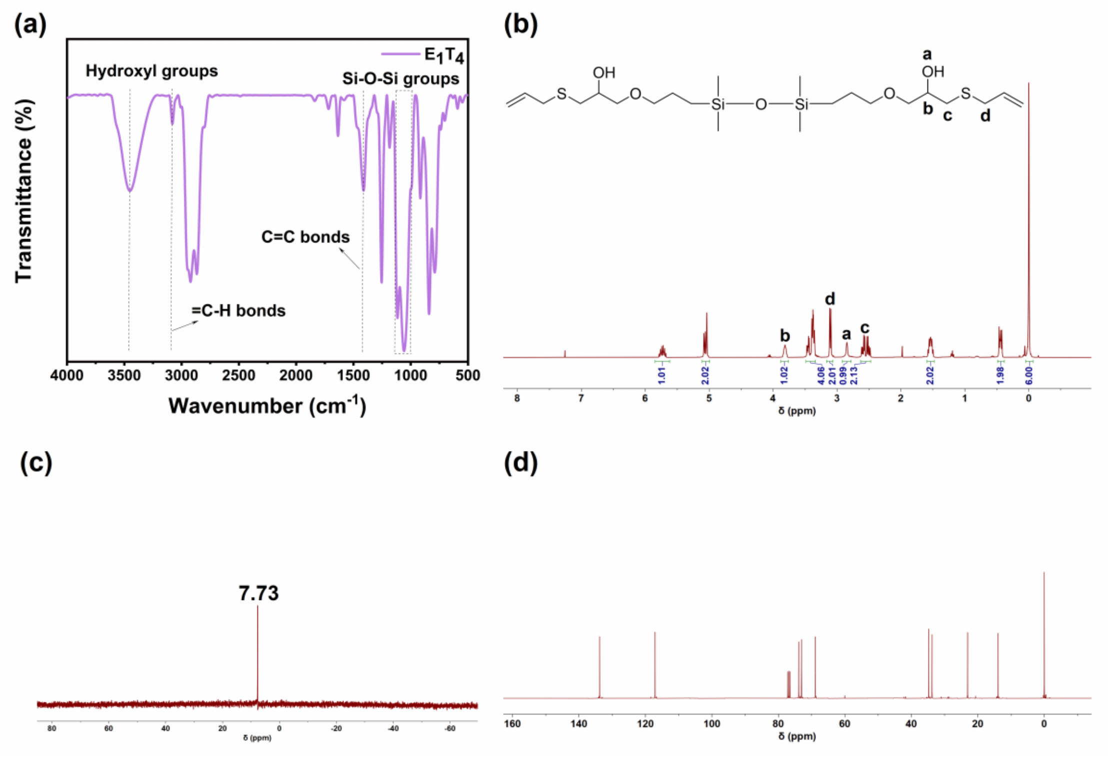

Figure S5. (a) FT-IR spectra of functionalized disiloxane E_1T_4; (b) ^1H NMR spectrum of E_1T_4; (c) ^{29}Si NMR spectrum of E_1T_4; (d) ^{13}C NMR spectrum of E_1T_4.

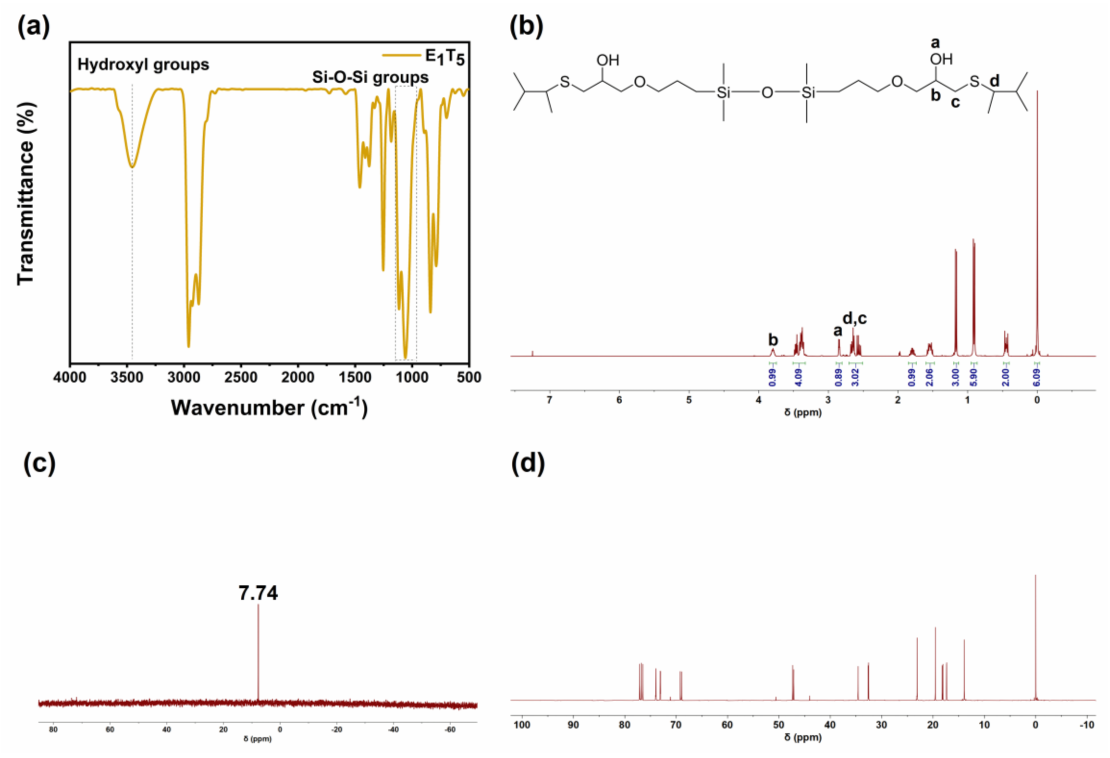

Figure S6. (a) FT-IR spectra of functionalized disiloxane E_1T_5; (b) ^1H NMR spectrum of E_1T_5; (c) ^{29}Si NMR spectrum of E_1T_5; (d) ^{13}C NMR spectrum of E_1T_5.

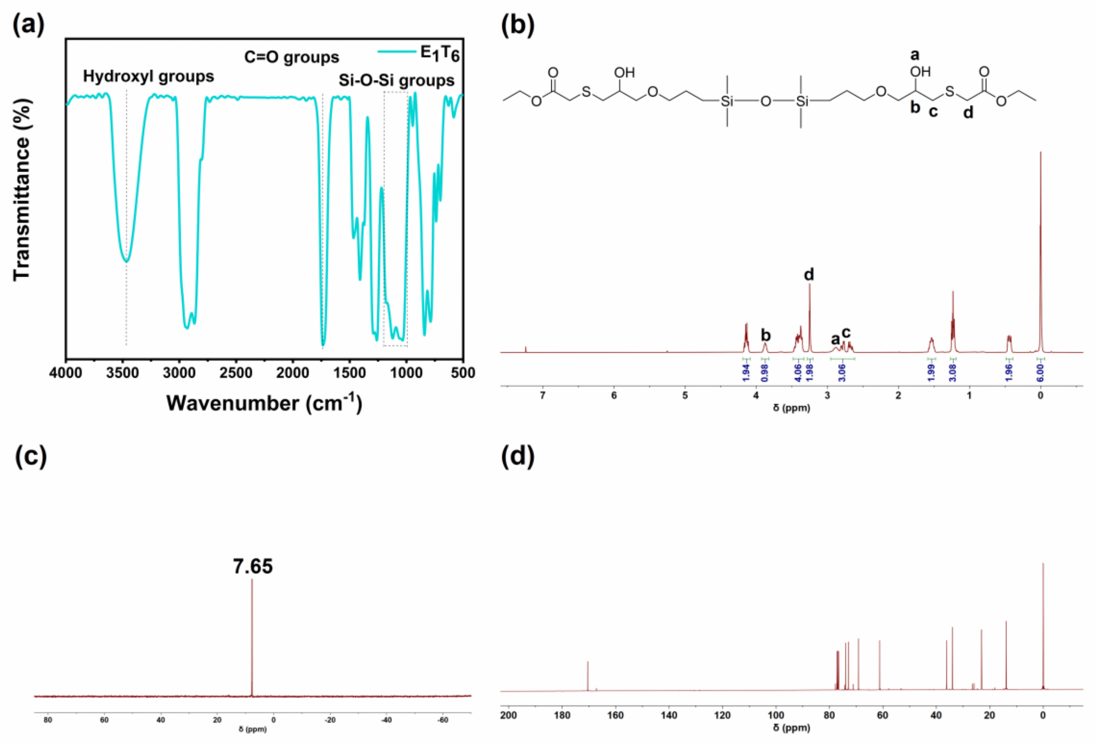

Figure S7. (a) FT-IR spectra of functionalized disiloxane E_1T_6; (b) ^1H NMR spectrum of E_1T_6; (c) ^{29}Si NMR spectrum of E_1T_6; (d) ^{13}C NMR spectrum of E_1T_6.

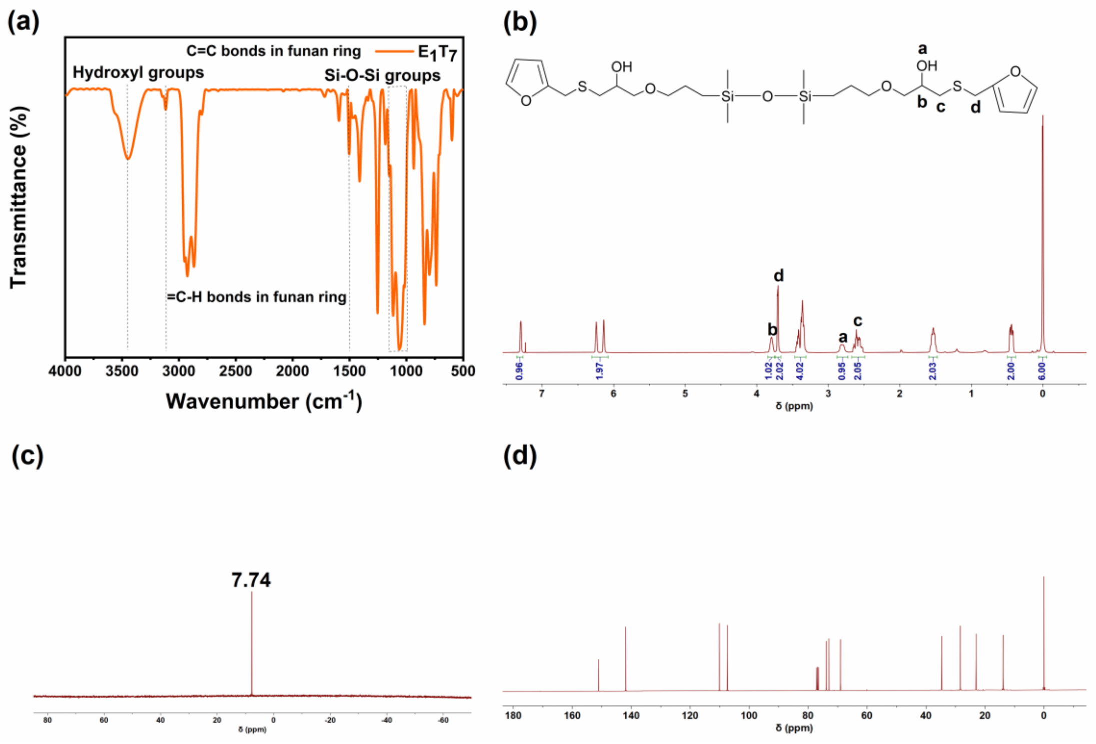

Figure S8. (a) FT-IR spectra of functionalized disiloxane E_1T_7; (b) ^1H NMR spectrum of E_1T_7; (c) ^{29}Si NMR spectrum of E_1T_7; (d) ^{13}C NMR spectrum of E_1T_7.

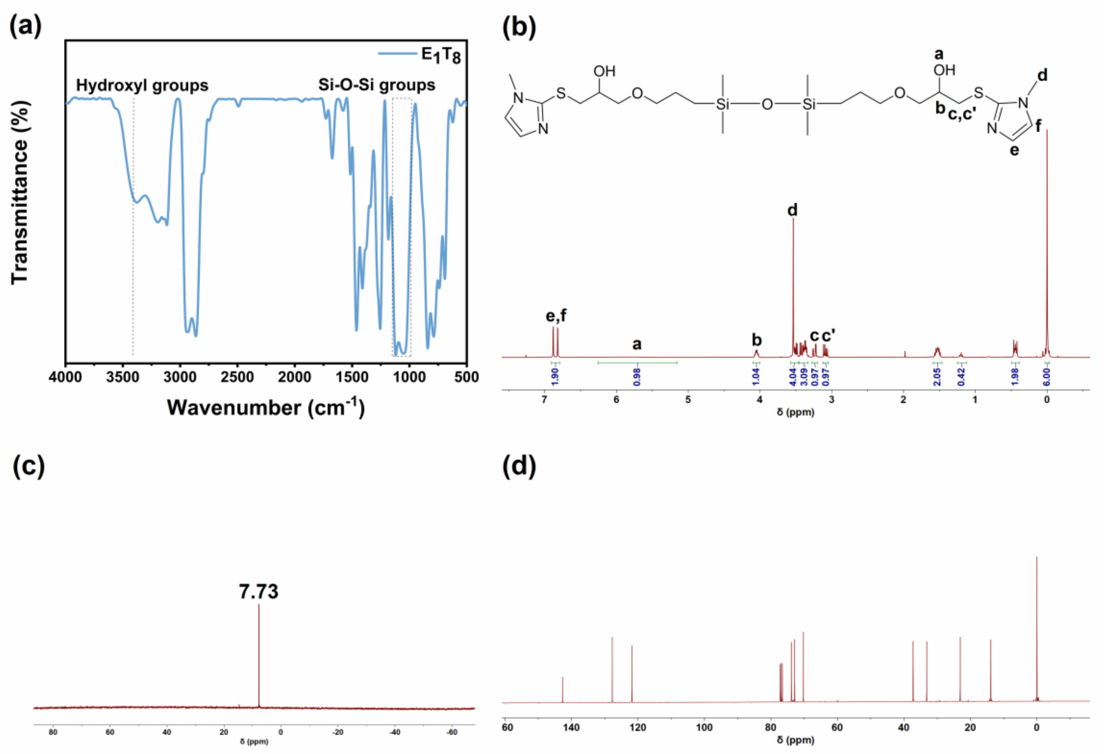

Figure S9. (a) FT-IR spectra of functionalized disiloxane E_1T_8; (b) ^{1}H NMR spectrum of E_1T_8; (c) ^{29}Si NMR spectrum of E_1T_8; (d) ^{13}C NMR spectrum of E_1T_8.

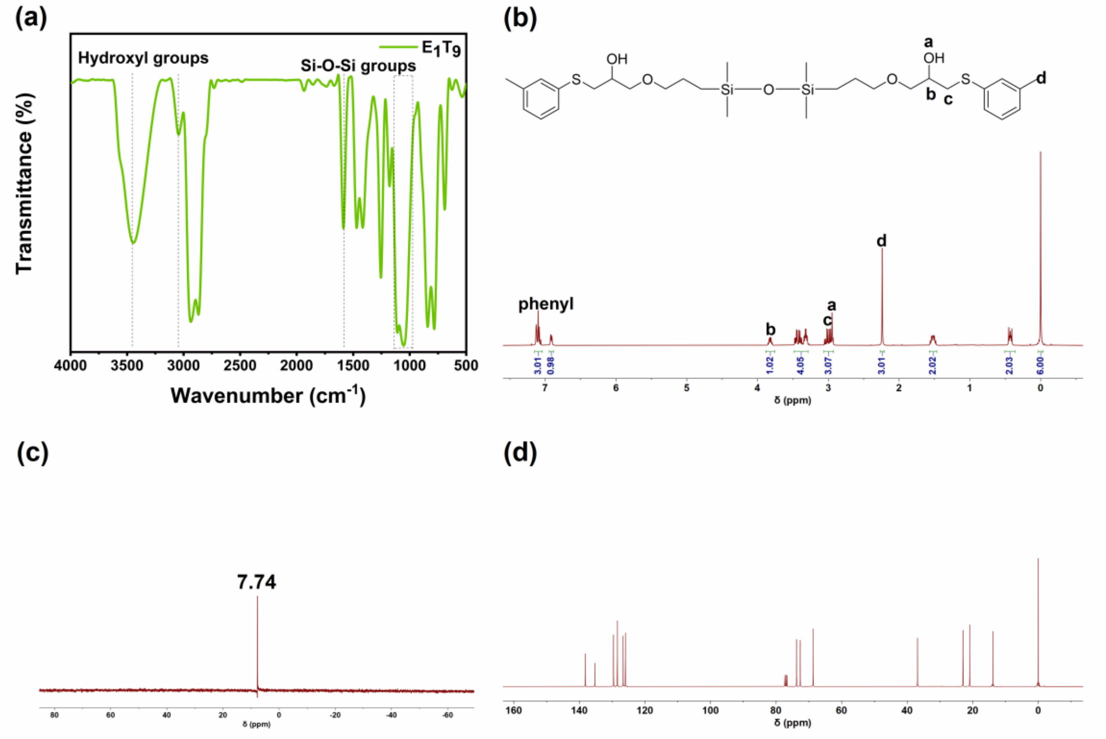

Figure S10. (a) FT-IR spectra of functionalized disiloxane E_1T_9; (b) ^{1}H NMR spectrum of E_1T_9; (c) ^{29}Si NMR spectrum of E_1T_9; (d) ^{13}C NMR spectrum of E_1T_9.

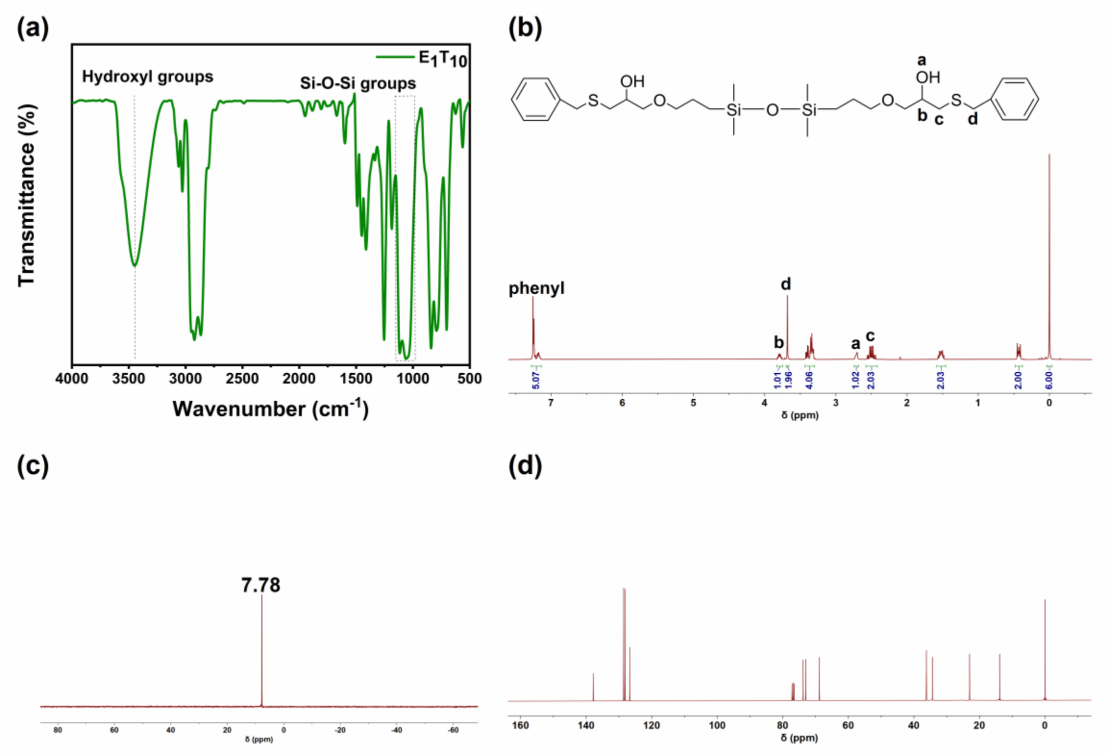

Figure S11. (a) FT-IR spectra of functionalized disiloxane E_1T_{10}; (b) ^1H NMR spectrum of E_1T_{10}; (c) ^{29}Si NMR spectrum of E_1T_{10}; (d) ^{13}C NMR spectrum of E_1T_{10}.

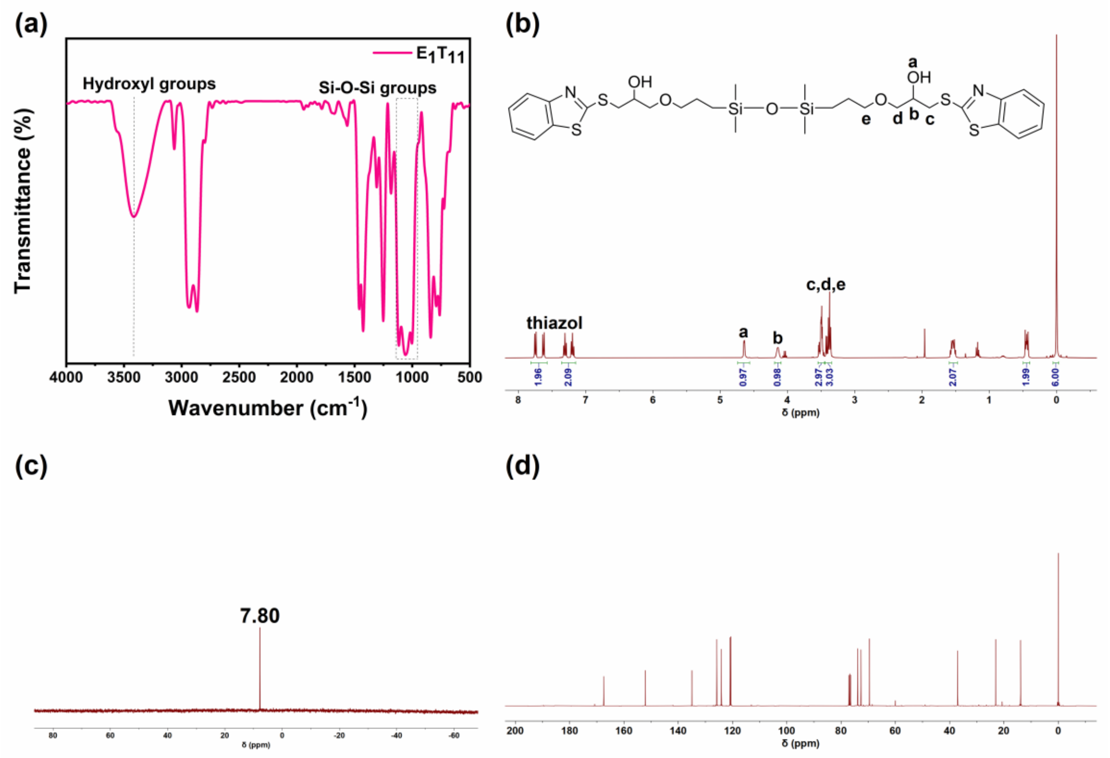

Figure S12. (a) FT-IR spectra of functionalized disiloxane E_1T_{11}; (b) ^1H NMR spectrum of E_1T_{11}; (c) ^{29}Si NMR spectrum of E_1T_{11}; (d) ^{13}C NMR spectrum of E_1T_{11}.

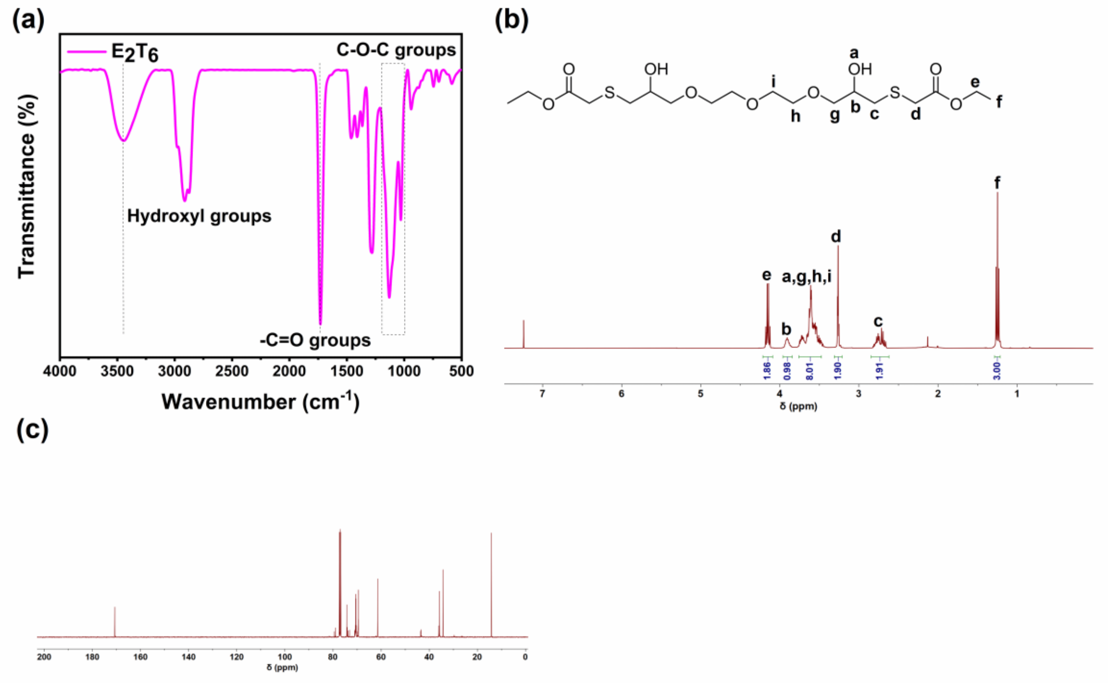

Figure S13. (a) FT-IR spectra of functionalized disiloxane E_2T_6; (b) ^{1}H NMR spectrum of E_2T_6; (c) ^{13}C NMR spectrum of E_2T_6.

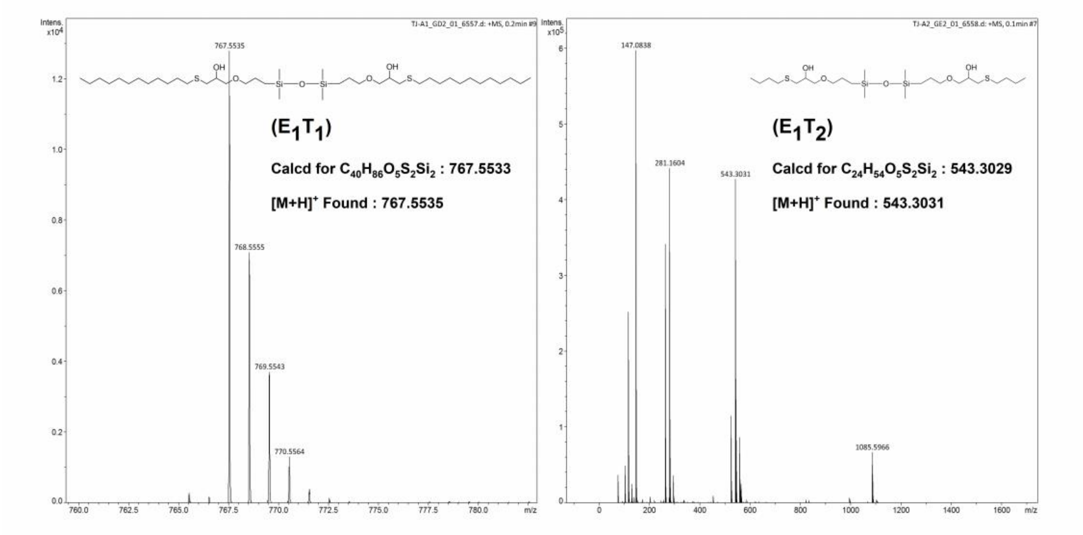

Figure S14. HR-MS spectra of functionalized disiloxane E_1T_1 and E_1T_2

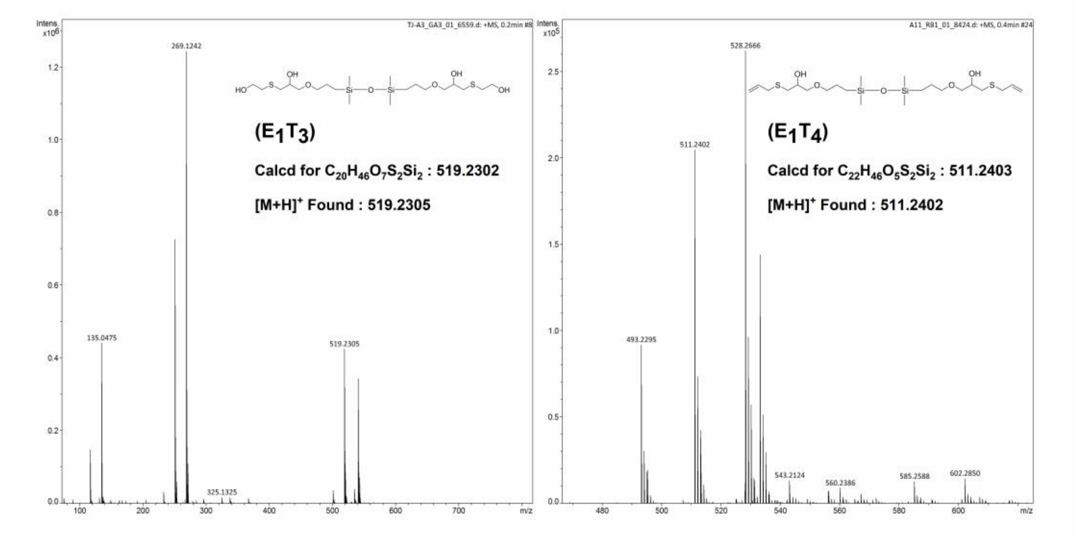

Figure S15. HR-MS spectra of functionalized disiloxane E_1T_3 and E_1T_4.

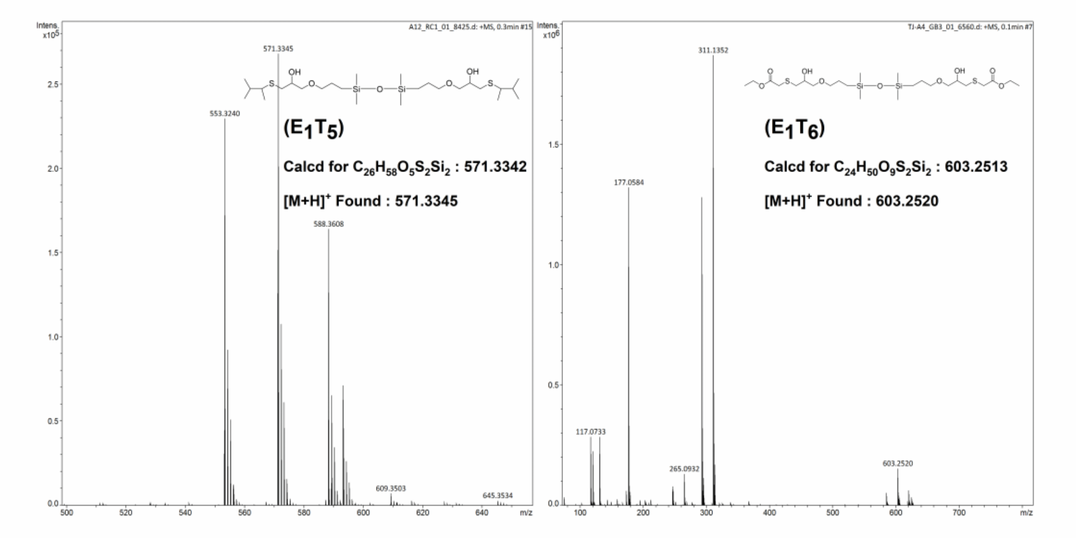

Figure S16. HR-MS spectra of functionalized disiloxane E_1T_5 and E_1T_6

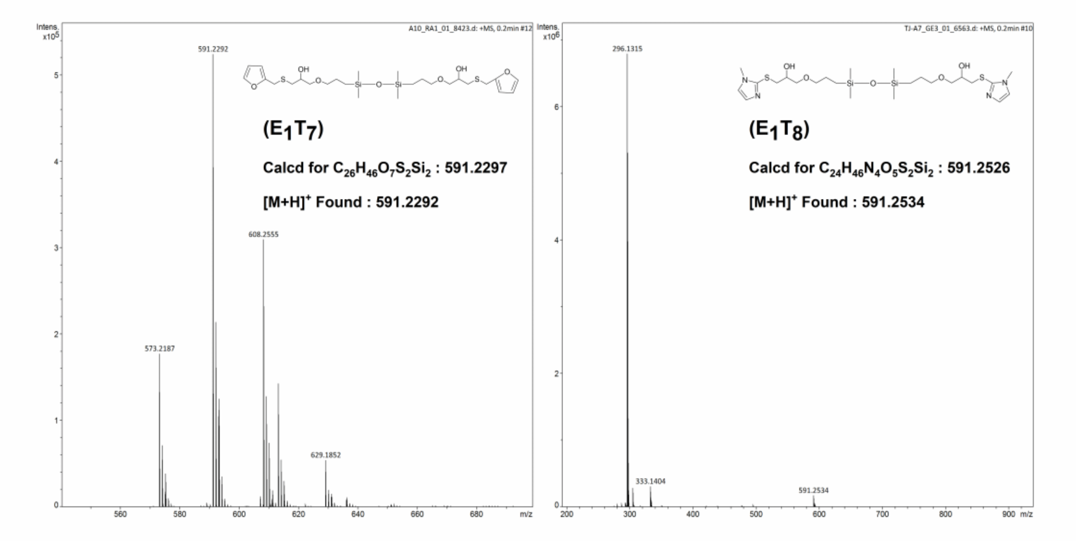

Figure S17. HR-MS spectra of functionalized disiloxane E_1T_7 and E_1T_8

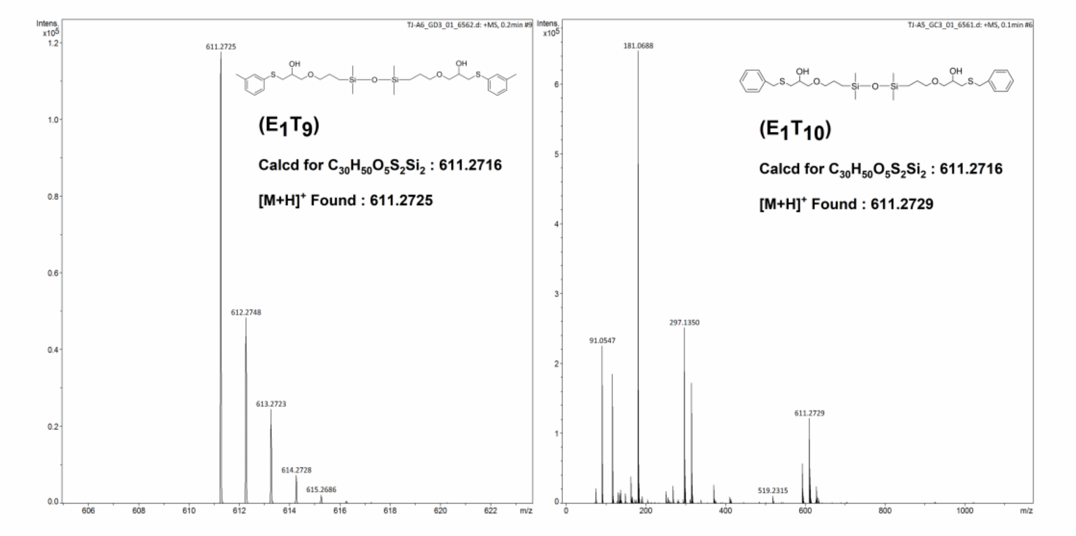

Figure S18. HR-MS spectra of functionalized disiloxane E_1T_9 and E_1T_{10}

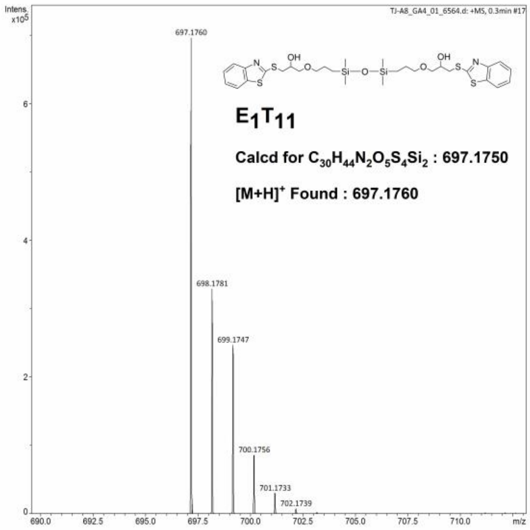

Figure S19. HR-MS spectra of functionalized disiloxane E_1T_{11}

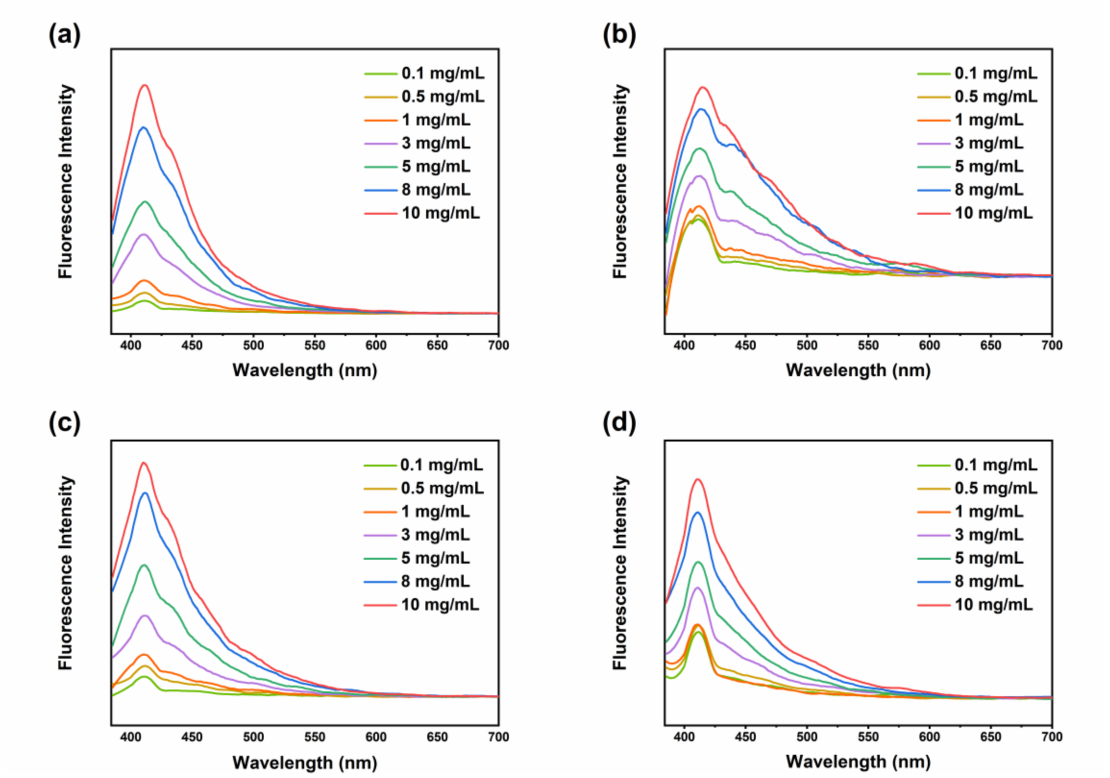

Figure S20. Fluorescence emission spectra of E_1T_1 (a), E_1T_2 (b), E_1T_3 (c), and E_1T_4 (d) with different concentration excited at 365 nm

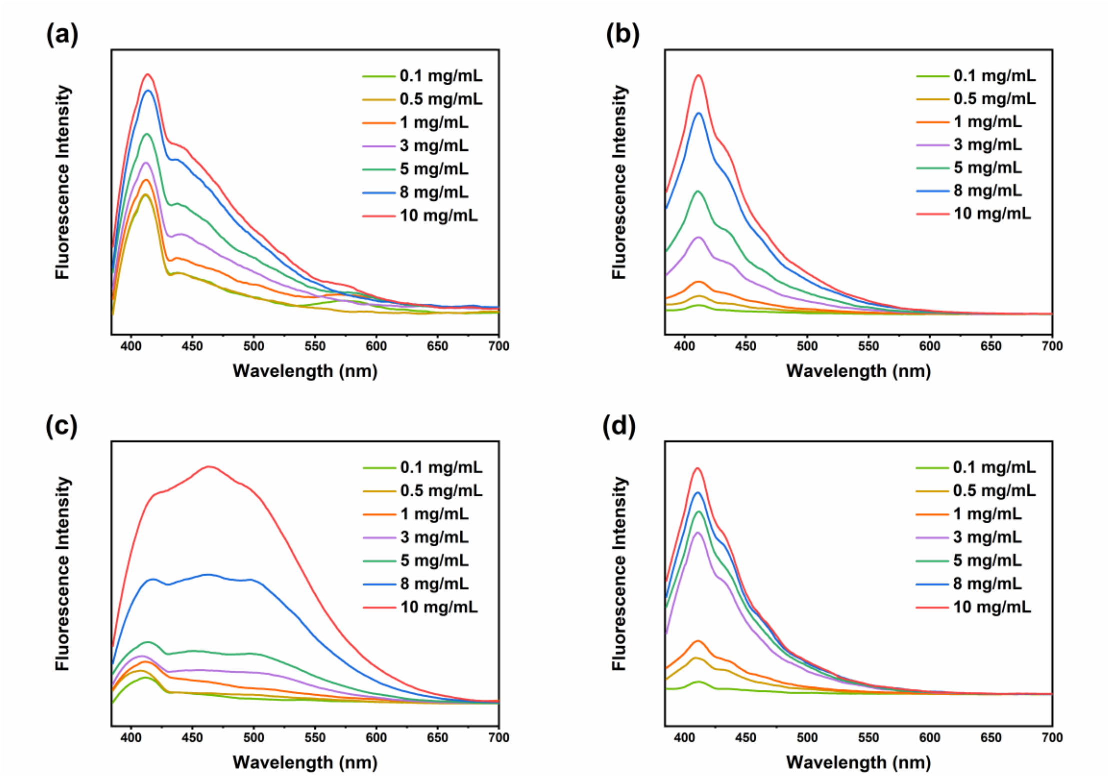

Figure S21. Fluorescence emission spectra of E_1T_5 (a), E_1T_6 (b), E_1T_7 (c), and E_1T_8 (d) with different concentration excited at 365 nm

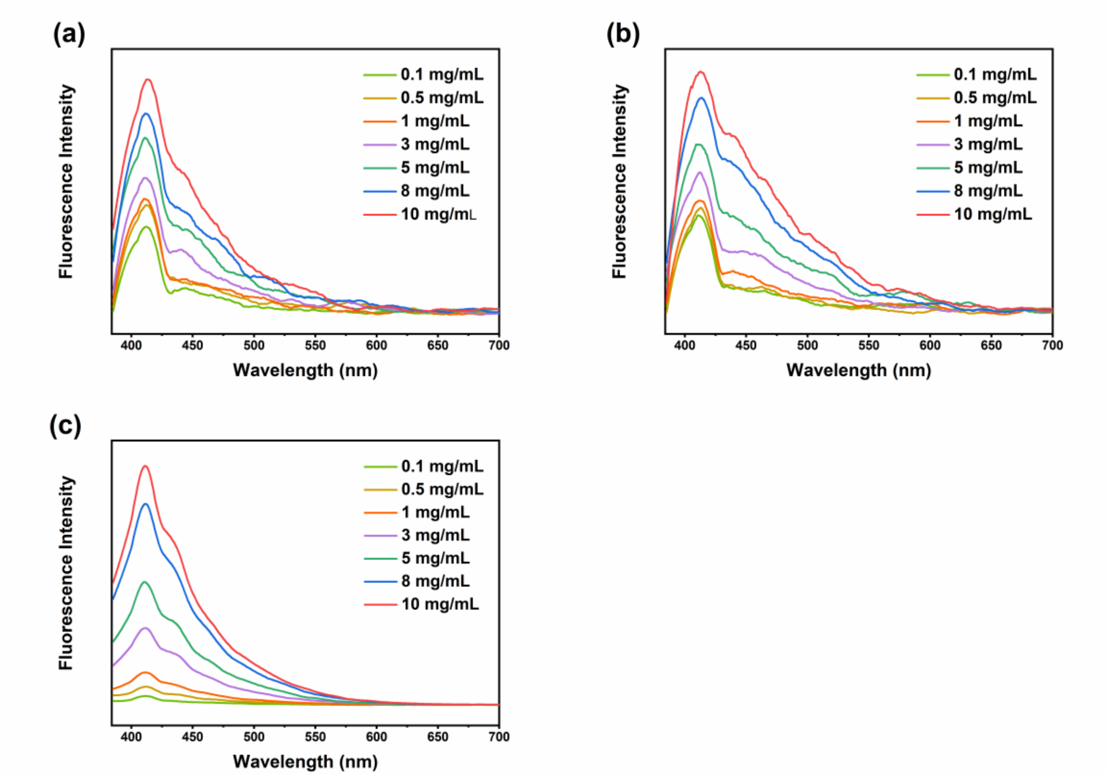

Figure S22. Fluorescence emission spectra of E_1T_9 (a), E_1T_{10} (b), and E_1T_{11} (c) with different concentration excited at 365 nm

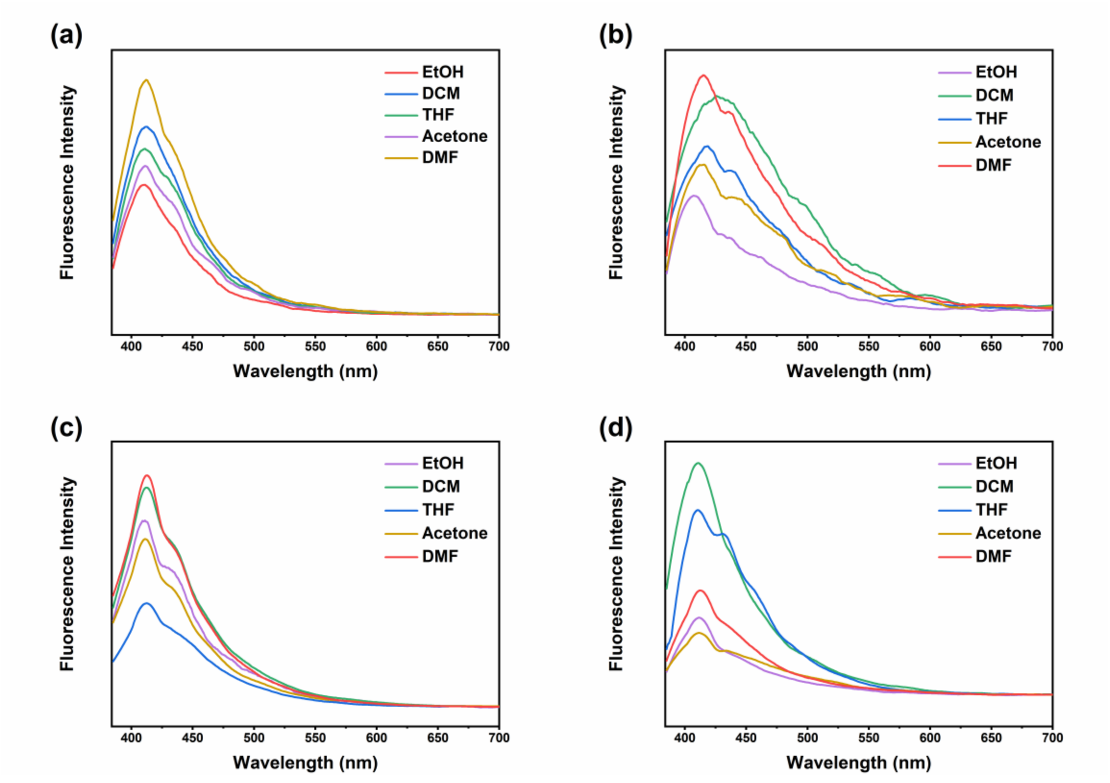

Figure S23. Fluorescence emission spectra of E_1T_1 (a), E_1T_2 (b), E_1T_3 (c), and E_1T_4 (d) in different solvents excited at 365 nm

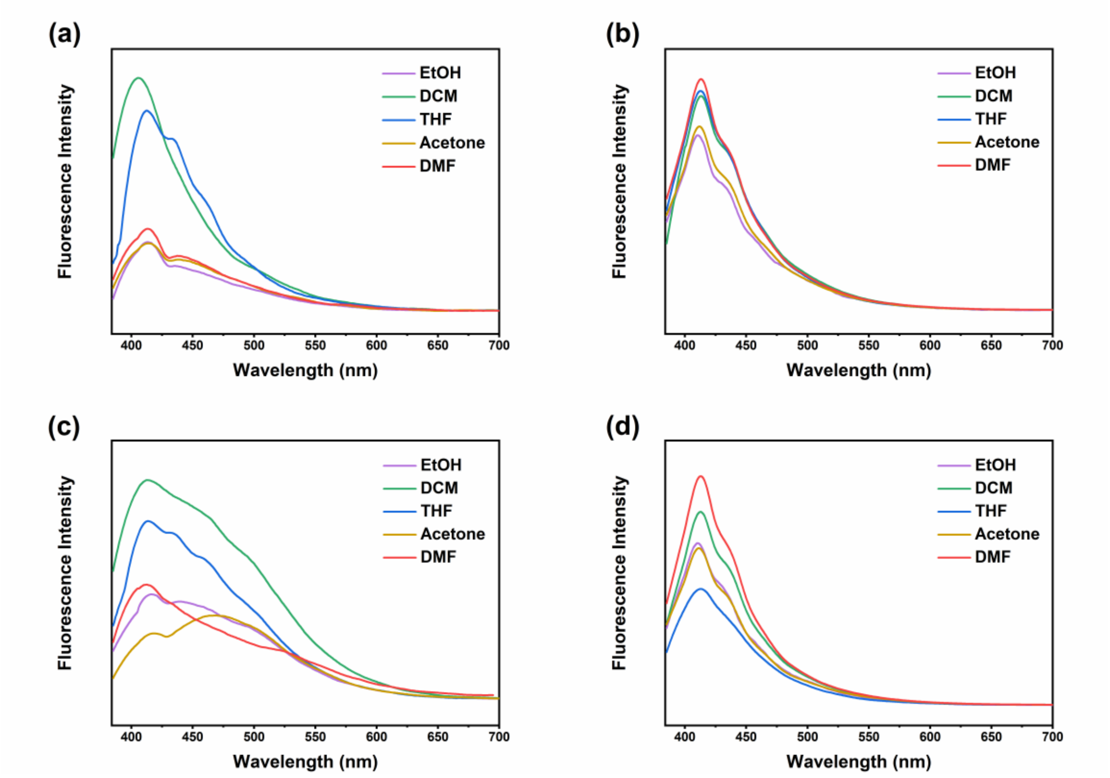

Figure S24. Fluorescence emission spectra of E_1T_5 (a), E_1T_6 (b), E_1T_7 (c), and E_1T_8 (d) in different solvents excited at 365 nm

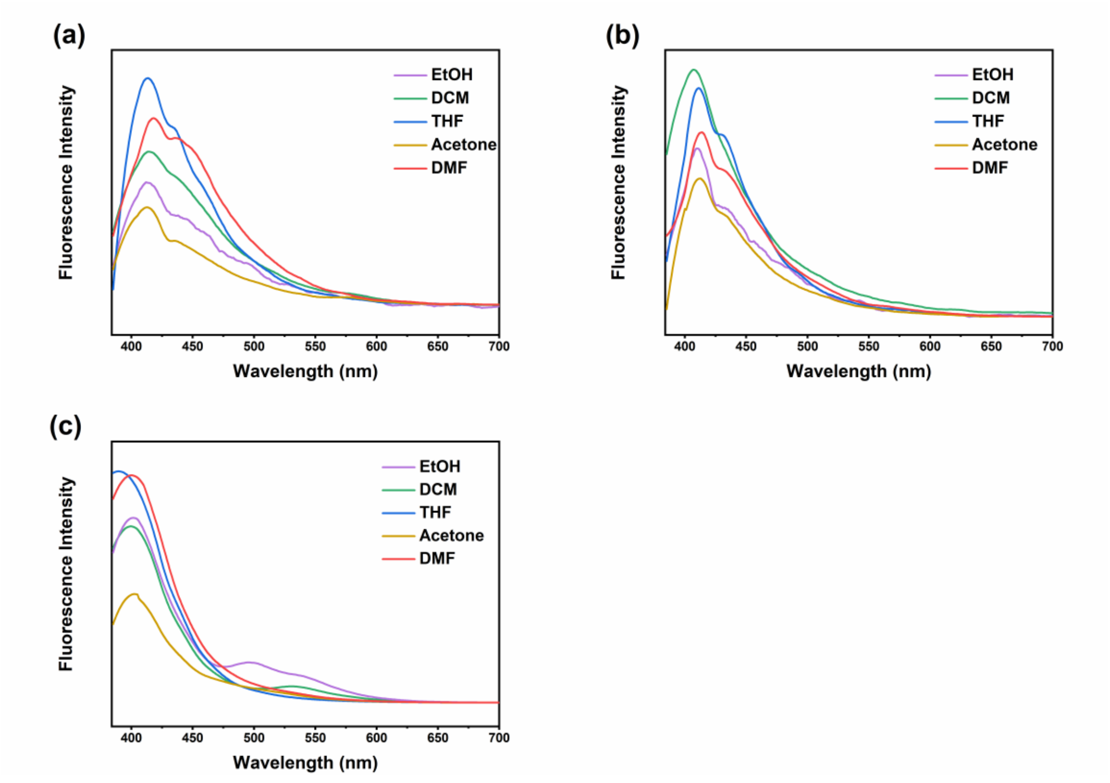

Figure S25. Fluorescence emission spectra of E_1T_9 (a), E_1T_{10} (b), and E_1T_{11} (c) in different solvents excited at 365 nm
